# Adsorption of Zwitterionic and Capped Amino Acids to Graphene: A Molecular Dynamics Study

**DOI:** 10.1021/acsomega.5c13030

**Published:** 2026-06-05

**Authors:** Antryg Benedict, Hui Li, Qi Yuan, Jason C. Bartz, Wei Zhang

**Affiliations:** † Department of Plant, Soil and Microbial Sciences, 3078 Michigan State University, East Lansing, Michigan 48824, United States; ‡ School of Medicine, Medical Microbiology, and Immunology, Creighton University, Omaha, Nebraska 68178, United States; § Environmental Science and Policy Program, Michigan State University, East Lansing, Michigan 48824, United States

## Abstract

Pyrogenic carbon (PyC) materials are commonly used to immobilize environmental contaminants and could also bind amino acids or proteins (including proteinaceous contaminants), reducing their availability to plants and other organisms. This study determined the adsorption free energies of 20 amino acids to graphene (model PyC surface) and explored the effects of ionic strength on adsorption and the adsorption mechanisms, using umbrella sampling molecular dynamics. We simulated the interactions of amino acids with graphene in their zwitterionic and capped forms at ionic strength of 0 and 0.5 M (NaCl), and further simulated the interactions of tryptophan, tyrosine, arginine, and lysine at ionic strength of 0.1, 0.2, and 0.3 M (NaCl). The aromatic amino acids and arginine adsorbed to graphene most strongly with adsorption free energies in the range of −20 to −40 kJ/mol. The adsorption free energies of the capped amino acids were on average 12.6 ± 2.8 kJ/mol more negative than those of the zwitterionic amino acids. Ionic strength had significant effects on the adsorption of capped aspartic acid, arginine, and tryptophan, despite generally insignificant effects when averaged across all amino acids. This study provided insights into the fate and transport of amino acids and proteins in the environment.

## Graphical abstract

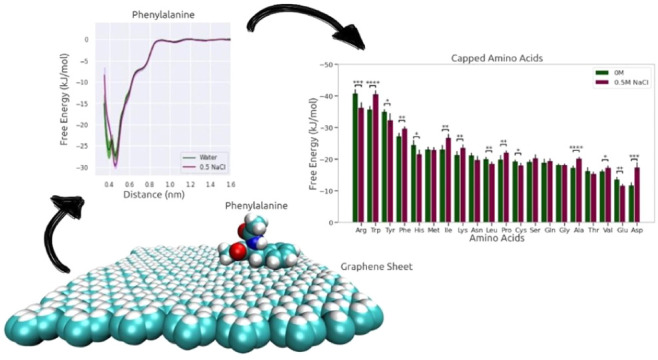

## 1 Introduction

Pyrogenic carbon (PyC) materials such as biochar and activated carbon are popular sorbents for immobilizing many environmental contaminants such as heavy metals, pesticides, pharmaceuticals, and polycyclic aromatic hydrocarbons due to PyC’s properties such as relatively high surface area, wide pore size distribution, and high aromatic carbon content.^1–5^ PyC can also adsorb proteins,^6,7^ which could influence the fate and activity of extracellular proteins (e.g., extracellular enzymes) and subsequently their ecosystem functions.^8^ Further, PyC may be effective in binding protein contaminants such as proteinaceous infectious particles (prions), thus reducing their mobility and infectivity in the environment.^9–15^

Proteins and free amino acids are major components of natural organic matter and make up approximately 18% of soil organic carbon and 40% of soil organic nitrogen.^16–18^ Proteinaceous matter also makes up 1–3% of dissolved organic carbon and 15–50% of dissolved organic nitrogen in surface waters, with concentrations between 2 and 6000 μg/L.^19–21^ Thus, studying the adsorption of proteins to PyC can provide insights into the cycling of carbon and nitrogen in the environment, as well as the fate and transport of extracellular enzymes and proteinaceous contaminants.

Proteins are composed of amino acids that are linked together to form polypeptide chains. In addition to serving as building blocks of proteins, amino acids can exist freely in their zwitterionic form, as illustrated by Supporting Information Figure S1. Protein adsorption to PyC and other surfaces is largely controlled by the interactions of their amino acid residues with the surfaces. While amino acids are largely found in the environment in combined forms (proteins, peptides, etc.),^19^ free amino acids can still play significant roles in the environment. For example, arginine is a source of nitrogen for plants and microorganisms and is a precursor for plant production of signal molecules related to stress response.^22,23^ Tryptophan is a precursor for plant growth hormones and can greatly influence plant growth and yield.^24–28^ Therefore, it is valuable to study the surface binding of amino acids in their free, zwitterionic form as well as in a capped form that more closely approximates amino acids in a protein chain environment by forming two peptide bonds and removing the charged termini.^29,30^

Computational approaches, such as molecular dynamics, are used to gain atomic-scale insight into adsorption processes.^31–33^ The adsorption of amino acids to surfaces is often studied to better understand the mechanisms of protein adsorption at the individual amino acid level, albeit excluding the effects of protein conformation. The adsorption free energies between amino acids and surfaces, including gold,^34,35^ titania,^36^ quartz,^37,38^ graphene,^29,39–49^ and graphene oxide,^50,51^ have been studied using molecular dynamics methods, and the results show good agreement with available experimental data. Previous studies of amino acid adsorption to graphene have largely used nonelectrostatic models for graphene, where interactions between amino acids and graphene were modeled using only van der Waals forces.^29,39,40,42–45,47,48^ While some results agree reasonably well with the limited experimental data available to date (Table S1 in the Supporting Information), those studies used an incomplete description of graphene, which has electrostatic properties due to the delocalized electrons on its surface.^52,53^ Dasetty et al. compared several different nonpolarizable force fields for amino acid adsorption to graphene and found the strongest adsorption of the aromatic amino acids and arginine, which is in accordance with the available experimental data.^39^ Another study by Hughes and Walsh modeled graphene using their own polarizable force field, GRAPPA.^41^ However, that study found anomalously strong binding for glutamine, glycine, and asparagine, which is inconsistent with the available experimental results.^41,54–56^ A more recent study by Soni et al. modeled amino acid adsorption to graphene using the CHARMM Drude Polarizable force field, which explicitly incorporates induced polarizability using charged particles that are attached to the atoms via harmonic oscillators.^46,57^ They found that arginine (a cationic amino acid) and the anionic amino acids, aspartic acid and glutamic acid, adsorbed most strongly to the graphene surface, which is also inconsistent with the available experimental results reporting the strongest adsorption for aromatic amino acids and arginine and only minimal adsorption of the anionic amino acids.^46,54–56^ Clearly there is still an important need to simulate the adsorption of amino acids to graphene.

The experimental studies on amino acid adsorption to carbon nanotubes and graphene suggest that the aromatic amino acids (tryptophan, tyrosine, phenylalanine, and histidine) and arginine bind most strongly, along with methionine and cysteine, whereas glycine, glutamine, asparagine, aspartic acid, and glutamic acid bind weakly.^54,56^ An experimental study on amino acid adsorption to graphene oxide found similar results, where they failed to detect the binding of glycine and aspartic acid and detected the most binding of aromatic and cationic amino acids, with the latter due to the presence of negatively charged oxide groups on the graphene oxide surface.^55^ Therefore, previously used polarizable force fields have not been able to produce results comparable to experimental studies of amino acid interactions with graphene, despite being a more accurate model of graphene’s properties. It is of interest to assess whether a different force field that incorporates graphene’s electrostatic properties, such as the Interface force field (IFF), can accurately model adsorption processes. IFF models graphene’s delocalized π electrons by adding “virtual electrons” above and below the graphene plane, whose distance from the carbon atoms is constrained, unlike the harmonic oscillators of the Drude Polarizable FF.^52,57^ Therefore, the IFF force field is not a true polarizable force field, but is able to more explicitly account for graphene’s electrostatic properties compared to force fields that model graphene using only van der Waals forces. It is also less computationally expensive compared to polarizable force fields.^52^ While IFF has accurately simulated carbon nanotube dispersion and solubility in water, the force field has not been assessed for its ability to model amino acid adsorption to graphene.^52^

The effects of ionic strength on the adsorption of amino acids to graphene were not explored in previous studies. Ionic strength can vary widely in the environment and can influence adsorption processes through the modification of electrostatic interactions and through salting-in/salting-out phenomena.^58–60^ Therefore, it is important to investigate the effects of ionic strength on amino acid adsorption to graphene using a molecular dynamics force field that explicitly accounts for the electrostatic properties of graphene.

In this study, we assessed the adsorption of the 20 natural amino acids to graphene in both their zwitterionic and capped forms using umbrella sampling molecular dynamics to estimate the free energies of adsorption at ionic strengths of 0–0.5 M (NaCl). This study aimed to assess whether the IFF for graphene is suitable for simulating amino acid/protein-graphene interactions, compare the adsorption of zwitterionic and capped amino acids, and assess the effect of solution ionic strength on amino acid adsorption to graphene. Adsorption of free, zwitterionic amino acids and capped amino acids to graphene has been studied only once by Dragneva et al.,^29^ which calculated the adsorption enthalpy rather than the free energy. Thus, this is the first study to compare the free energies of adsorption of the amino acids in their zwitterionic and capped forms to those of graphene. This study provides further insights into the interactions of proteins and amino acids with PyC in the environment.

## 2 Methods

### 2.1 Simulation System Preparation

Molecular dynamics umbrella sampling simulations were used to determine the free energies of adsorption of the 20 common amino acids on graphene. We also investigated the effects of ionic strength on the free energies of adsorption by running simulations at 0 and 0.5 M NaCl. Further umbrella sampling simulations were conducted at 0.1, 0.2, and 0.3 M NaCl with the amino acids tryptophan (Trp), tyrosine (Tyr), arginine (Arg), and lysine (Lys) to explore the effects of ionic strength on the adsorption of aromatic (Trp and Tyr) and cationic (Arg and Lys) amino acids to graphene.

To set up the system, a single-layer graphene sheet was generated using CHARMM-GUI Nanomaterial Modeler, with dimensions of 3.45 × 3.42 nm, periodic in the *X* and *Y* directions.^61,62^ The amino acids were modeled using PyMOL and acetyl (ACE) and *N*-methylamide (NMA) groups were added to the N and C termini, respectively, to prepare the capped amino acids (Supporting Information Figure S1).^63^ Graphene was modeled using the InterfaceFF (IFF) virtual electron force field, which explicitly incorporates graphene’s pi electron density and electrostatic properties, unlike the CHARMM and AMBER force fields used in the previous studies of amino acid adsorption to graphene.^29,39,40,52^ CHARMM36m and CHARMM-modified TIP3P water were used for the amino acids, ions, and water.^64,65^ This combination of force fields is fully compatible and can reproduce the interfacial properties of the graphene–water interface to experimental accuracy.^52^

GROMACS 2022 was used for setting up, running, and analyzing the simulations, and visualizations were performed with VMD.^66–68^ In each simulation, the amino acid was inserted above the graphene sheet with a random orientation in the simulation box using the GROMACS tool insert-molecules and the system was solvated with ∼2200–2250 water molecules. Systems with a net nonzero charge were neutralized using sodium or chloride counterions. Additional Na^+^ and Cl^–^ ions were added for simulations at 0.5 M NaCl. Simulations at 0.1, 0.3, and 0.5 M NaCl were also performed for the zwitterionic and capped forms of Trp, Tyr, Arg, and Lys. A box size of ∼3.4 × 3.4 × 8.4 nm was used for the 0 M simulations, incorporating a vapor region of approximately 1 nm at the top and bottom of the box, following the approach used in the amino acid-graphene force field benchmark study by Dasetty et al. (Figure 1).^39^ The vapor region was removed for the simulations at higher ionic strength to avoid possible artifacts from ion interactions with the air–water interface (Figure 1).^69,70^

**Figure 1:**
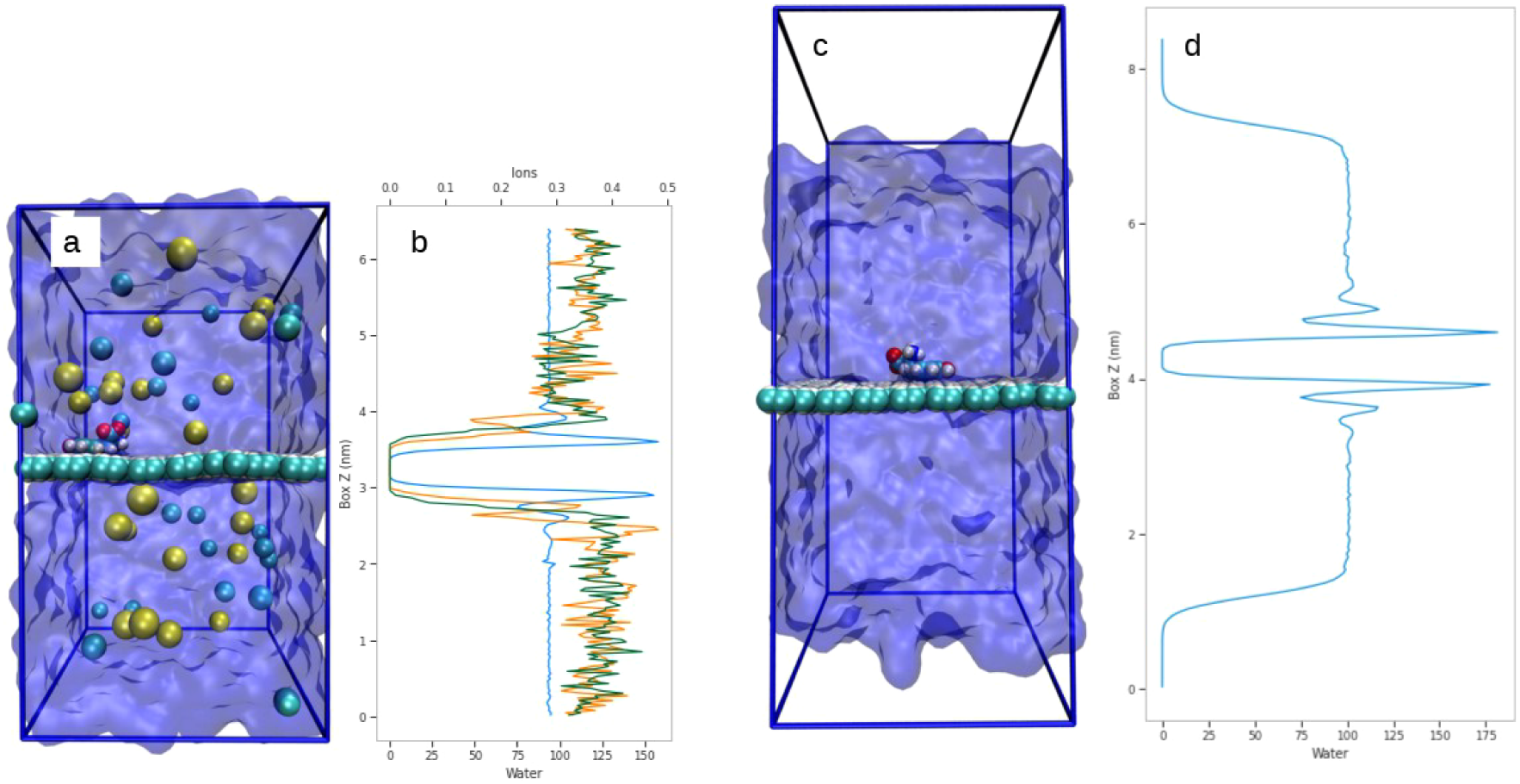
Simulation boxes with (a) and without (c) 0.5 M NaCl for zwitterionic tyrosine as an example. Number density plots (b for simulation box a and d for simulation box c): blue is water, orange is Na^+^, and green is Cl^–^.

All systems were energy-minimized using steepest descent to under 100 kJ/mol/nm. Bonds between hydrogen atoms and heavy atoms were constrained using the LINCS algorithm and water covalent bonds were constrained using the SETTLE algorithm.^71,72^ Systems were equilibrated at 300 K for 1 ns in the NVT ensemble using the v-rescale thermostat with separate temperature coupling groups for the amino acid, solvent, and graphene, with a time constant of 1.0 ps.^72^ The amino acid and graphene sheet were restrained using a force constant of 1000 kJ/(mol·nm^2^) during equilibration. An additional 500 ps NPT equilibration was carried out for systems at 0.1 M or higher ionic strengths to equilibrate the density to 1 bar using a Parrinello–Rahman semiisotropic barostat.^73^ We did not include this step for simulations that incorporated a vapor layer. All production simulations were in the NVT ensemble. Short-range electrostatics had a cutoff of 1.2 nm, and long-range electrostatics were calculated using particle-mesh Ewald.^74^ van der Waals forces were calculated using a cutoff of 1.2 nm with a force-switch at 1.0 nm. A time step of 2 fs was used.

### 2.2 Umbrella Sampling Simulations

The GROMACS pull code was used to perform umbrella sampling using the cylinder geometry option so that the graphene-amino acid distance was defined as the distance between the center of mass (COM) of the amino acid and the atoms of the graphene sheet within a radius of 0.8 nm around the COM of the amino acid. As such, the amino acid was restrained to a specific distance away from the graphene sheet in the *Z* direction, but was free to move in the *X* and *Y* directions.^67^ Twenty-three windows were used with amino acid–graphene distances from 0.4 to 2.0 nm. Windows between 0.4 and 0.8 nm had a spacing of 0.05 nm (amino acid-graphene distances of 0.4 nm, 0.45 nm, 0.5 nm, etc.) and a force constant of 8000 kJ/(mol·nm^2^). Windows between 0.9 and 2.0 nm had a spacing of 0.1 nm (amino acid-graphene distances of 0.9 nm, 1.0 nm, 1.1 nm, etc.) and a force constant of 4000 kJ/(mol·nm^2^). With this setup, we found good sampling overlaps between the windows (Supporting Information Figures S2–S9).

Umbrella sampling simulations were run for 10 ns with data collected every 10 ps for umbrella sampling distances and the last 7 ns were used for Potential of Mean Force (PMF) calculations. Five replicates were performed for each system. The GROMACS implementation of WHAM, g_wham, was used to calculate the PMFs using Bayesian bootstrapping with 100 bootstraps for each system, 200 bins (binning width of ∼0.0088 nm), and autocorrelations calculated.^75^ The adsorption free energy was calculated as the difference in energy between the minimum energy at adsorption and the energy when the graphene-amino acid distance was 1.5 nm.

We compared our results to two previous studies, i.e., Dasetty et al. that used CHARMM36 to model the graphene surface and Soni et al. that used the CHARMM Drude Polarizable force field for all components of their simulations.^39,46^ CHARMM36 models graphene as an uncharged surface and implicitly accounts for graphene’s delocalized electron density using the 12–6 Lennard-Jones potential.^39,64^ IFF uses negatively charged dummy atoms placed above and below the plane of the graphene sheet to more explicitly account for graphene’s delocalized electron density.^52^ The Drude Polarizable force field models the induced polarization of atoms using charged particles attached to the atoms via harmonic oscillators.^57^ The addition of more particles, as well as the shorter time step required for the harmonic oscillators, makes the Drude Polarizable force field significantly more computationally expensive than nonpolarizable force fields.

## 3 Results and Discussion

### 3.1 Effect of Graphene Force Fields

There are currently no experimental studies that have determined the free energies of adsorption for all 20 amino acids, and therefore we cannot quantitatively compare our results to the experimental data. Nonetheless, we have qualitatively compared our results to those reported in the literature. Adsorption of 16 amino acids to single-walled carbon nanotubes (SWNTs) followed the order of Phe > Tyr > Arg ∼ Met > Cys ∼ His > Leu ∼ Ile > Gln ∼ Asn > Val > Glu ∼ Lys ∼ Asp ∼ Ser ∼ Thr.^54^ A liquid chromatography study on amino acid adsorption to SWNTs found that the amino acids had retention times (surrogates of amino acid affinity to SWNTs) in the order of Trp ≫ Tyr ∼ His ∼ Phe > Ile ∼ Leu ∼ Val ∼ Gly ∼ Ala.^56^ Our results are largely in accordance with these experimental data, in which Trp, Tyr, Arg, Phe, and His are the five amino acids that adsorb more strongly than others. However, our simulations found that Arg adsorbed most strongly, followed by Trp > Tyr > Phe > His, which is different from the experimental results that reported the stronger adsorption of Phe and Tyr than Arg and even stronger adsorption of Trp than Tyr, Phe, and His. Both studies were conducted on carbon nanotubes, rather than flat graphene surfaces, which might contribute to the differences between our results and the experimental results. Molecular dynamics of a single amino acid adsorbing to a surface is also unable to account for possible interactions between neighboring amino acids adsorbed to the surface, which could influence the experimental results.

It is common in modeling studies to compare results obtained by various modeling approaches to assess uncertainty, the result range, and model suitability. We thus compared our results to two previous molecular dynamics studies: a study by Dasetty et al. that used a nonpolarizable force field for graphene (CHARMM36) and a more recent study by Soni et al. (Figure 2) that used the CHARMM Drude Polarizable Force field.^39,46^ The free energies of adsorption for our study and that of Dasetty et al. are similar for many of the amino acids. However, in this study, arginine (Arg), histidine (His), isoleucine (Ile), glycine (Gly), and alanine (Ala) have significantly stronger free energies of adsorption, and tryptophan (Trp), phenylalanine (Phe), methionine (Met), threonine (Thr), and glutamic acid (Glu) have significantly weaker free energies of adsorption compared to the results of Dasetty et al..^39^ Arginine’s side chain is positively charged, and the addition of the negatively charged virtual pi electrons to the graphene surface (IFF) results in an electrostatic attraction that was absent in Dasetty et al.. Thus, IFF may better reproduce cation-pi interactions between arginine and aromatic carbon rings, although further experimental validation is required.^77–80^ Lysine and histidine can also participate in cation-pi interactions. Histidine has a partial positive charge on its imidazole ring, which also allows for an electrostatic attraction between histidine and the graphene sheet. As with arginine, the positively charged side chain of lysine is able to form cation-pi interactions with the delocalized electrons of aromatic rings.^77^ This interaction is significant in protein folding, protein–protein interactions, and protein–ligand interactions, and can also contribute to protein binding to graphene and other aromatic carbon surfaces.^77–80^ However, we did not find a similarly stronger adsorption for lysine, the other cationic amino acid, likely due to differences in orientation, which will be discussed in the next section. Without experimentally determined adsorption free energies that can be quantitatively compared to simulation results, it is impossible to determine which force field, CHARMM36 or IFF, is a better model for graphene. Nonetheless, IFF is indeed shown to be able to account for interactions involving delocalized electrons.

**Figure 2:**
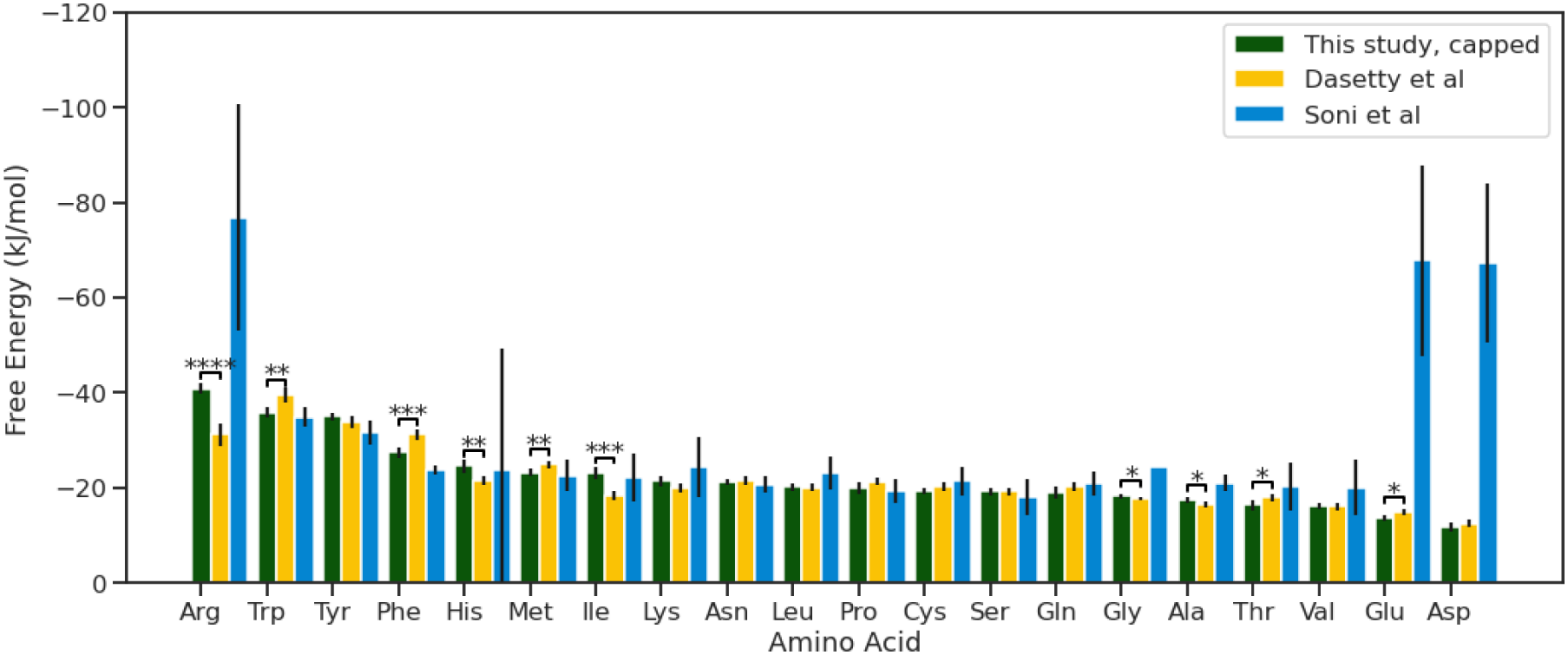
Comparison of adsorption free energies of capped amino acids to graphene in this study, Dasetty et al.,^39^ and Soni et al.^46^ Our study and that of Dasetty et al. used CHARMM36 for the amino acids. Dasetty et al. used CHARMM36 for graphene, whereas this study used IFF for graphene. Soni et al. used the CHARMM Drude Polarizable Force field for amino acids and graphene. Comparisons of the means of the simulations (5 replicates) were tested for our study and and that of Dasetty et al. using MedCalc^76^ with the significance level: *P* ≤ 0.0001 is “****”, *P* ≤ 0.001 is “***”, *P* ≤ 0.01 is “**”, and *P* ≤ 0.05 is “*”. Comparison of the means of the simulations was not possible with Soni et al.^46^

Our results and those of Soni et al.^46^ are also similar for most amino acids, except for arginine (Arg), aspartic acid (Asp), and glutamic acid (Glu), which had much more negative free energies of adsorption in Soni et al.. Soni et al. used a fully polarizable force field for all components of their simulations (graphene, amino acids, water, etc.), while we used IFF for graphene, a nonpolarizable force field that explicitly accounts for graphene’s delocalized electrons, and CHARMM36 for the amino acids.^46,52^ In principle the polarizable force field should produce more accurate results, due to incorporating the polarizability of the atoms. However, their study found much stronger adsorption of the anionic amino acids, aspartic acid and glutamic acid, especially compared to the aromatic amino acids, tryptophan, tyrosine, and phenylalanine. Experimental studies have shown that aromatic amino acids are far more likely to adsorb to carbon nanotubes, while anionic amino acids adsorbed very little if at all.^54,56^ Therefore, it is likely that the Drude Polarizable FF overestimates anion-graphene interactions, and may not be a suitable choice for studying amino acids/protein-graphene interactions.

### 3.2 Adsorption of Zwitterionic and Capped Amino Acids

Figure 3 and Supporting Information Table S2 show the adsorption free energies of the capped and zwitterionic amino acids at 0 and 0.5 M NaCl. The individual PMFs are provided in Supporting Information Figures S10 and S11. Adsorption free energies of capped amino acids were significantly more negative (*P*-value: 1.7 e-6), with an average difference of 12.6 ± 2.8 kJ/mol. This is likely due to the increased size and decreased charge density of the capped amino acids compared to their zwitterionic forms, which results in stronger van der Waals interactions and less favorable interactions with water, resulting in hydrophobic effects. This effect is especially significant for smaller amino acids such as glycine, serine, threonine, etc., where the capping groups make up a larger mass fraction of the amino acids

**Figure 3:**
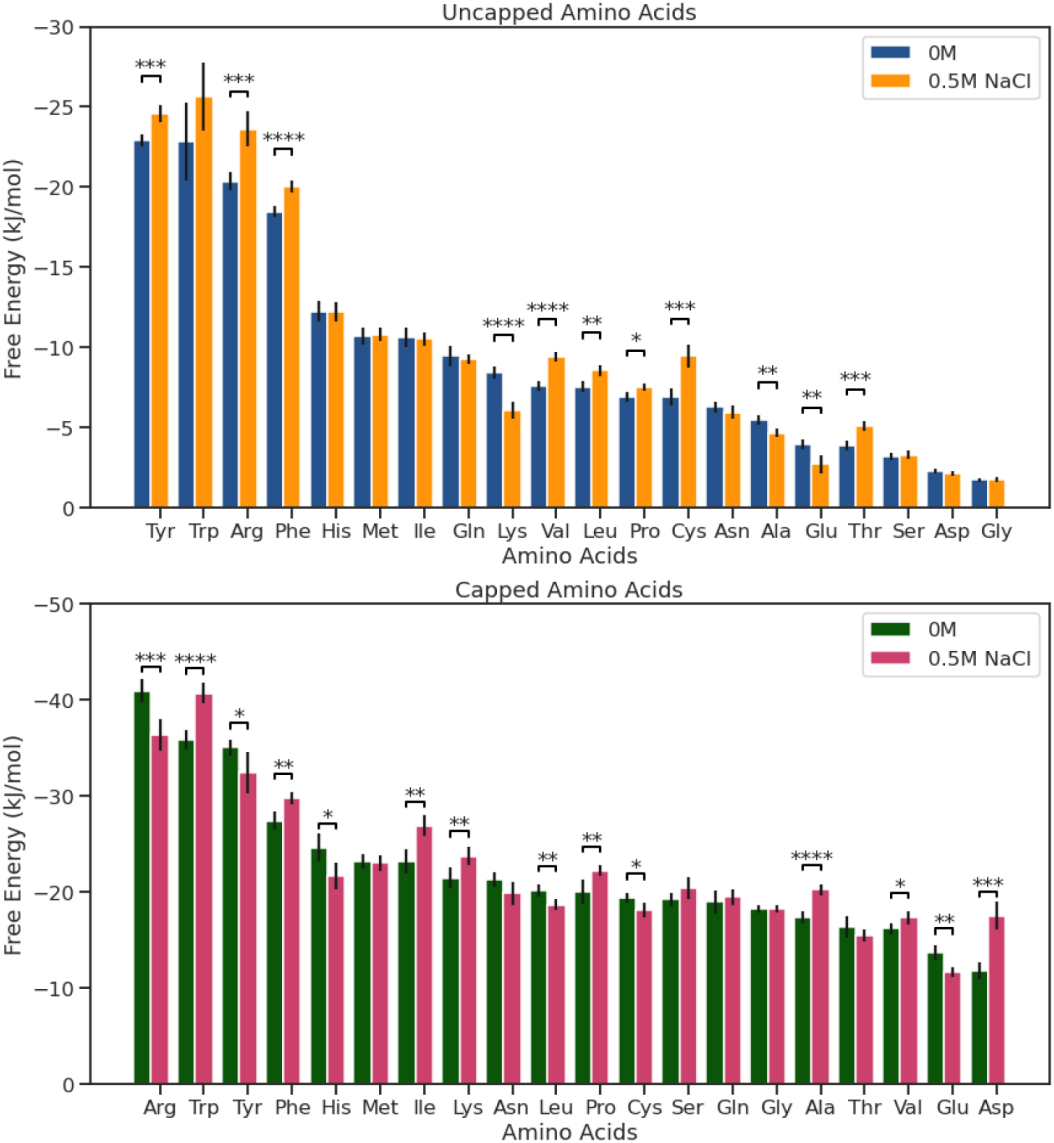
Free energies of adsorption of zwitterionic amino acids (upper) and capped amino acids (lower) in 0 and 0.5 M NaCl. The adsorption free energies were calculated as the difference in energy between the energy minimum and when the graphene and amino acids are fully separated (1.5 nm separation distance). Comparisons of the means of the simulations in 0 and 0.5 M NaCl (5 replicates) were tested using MedCalc^76^ with the significance level: *P* ≤ 0.0001 is “****”, *P* ≤ 0.001 is “***”, *P* ≤ 0.01 is “**”, *P* ≤ 0.05 is “*”, and no stars shown for insignificant differences.

Figure 4 shows the average distance (density profiles) of different parts of the amino acids during the last 7 ns of the 10 ns simulations for the windows where the free energy between the amino acid and graphene was the lowest (for most amino acids, this was the 0.3 nm window). While density profiles are generally calculated with unbiased simulations, in this case we were interested in determining the preferential orientation of the amino acids in the lowest energy windows. The amino acids were split into their side chain, α carbon, and their two termini: ACE and NME for the capped termini, and N^+^ and COO^–^ for the uncapped termini. When the distance between graphene and a part of the amino acids is around 0.3 nm or less, that part of the amino acid is in direct contact with the surface. For example, capped lysine’s backbone groups (ACE and NME) are closest to the surface, while the side chain is further away, indicating that capped lysine is oriented so the backbone is in contact with the surface and the side chain is pointed away from the surface.

**Figure 4:**
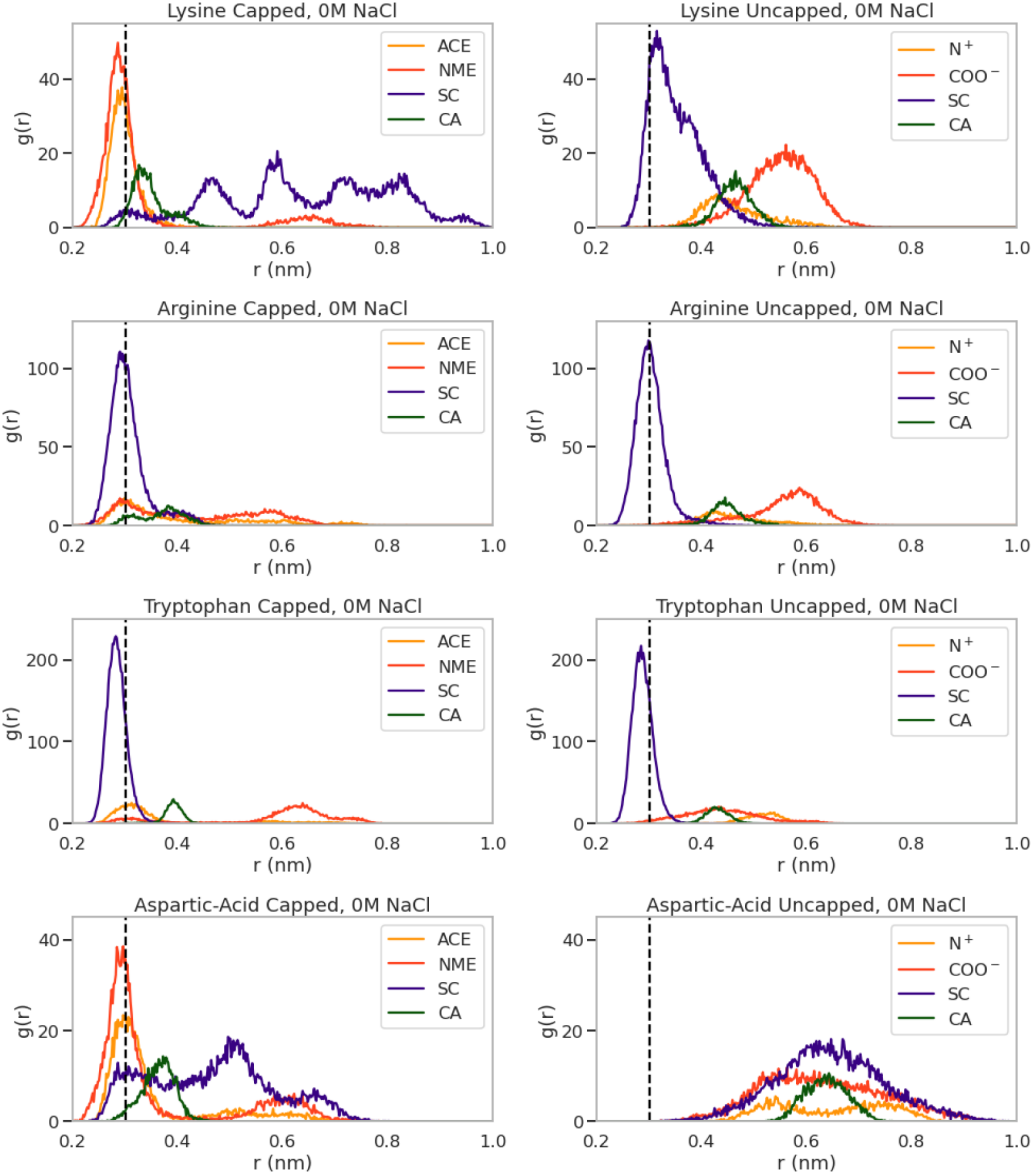
Density profiles: probability (*g*(*r*)) of finding parts of the amino acids at a given distance (*r*) from the graphene surface. We calculated the density profiles from the window where the free energy between the amino acids and the surface was the lowest. For most amino acids, this was around 0.3 nm distance from the graphene surface, but it was 0.6 nm for aspartic acid. This indicates the orientation of the amino acid relative to the surface. N^+^ and COO^–^ are the zwitterionic amino acid termini, ACE and NME are the capped amino acid termini, SC is the side chain, and CA is the α carbon. The dashed black line at 0.3 nm is the cutoff for direct contact; when atoms are within 0.3 nm of the graphene surface, they are in direct contact with it. The density profiles for all capped and zwitterionic amino acids are provided in Supporting Information Figures S12 and S13.

The density profiles for all amino acids are shown in Supporting Information Figures S12 and S13. These results indicate that the aromatic amino acids such as tryptophan adsorb to graphene through their aromatic side chains, in both their capped and zwitterionic forms. Arginine, a cationic amino acid, also binds to graphene through its side chain, likely because arginine’s side chain is large and planar, and because of favorable electrostatic interactions. Other capped amino acids adsorb to graphene through their capping groups/backbone, including lysine, the two anionic amino acids (glutamic acid and aspartic acid), two small and polar amino acids (serine and threonine), and alanine. The zwitterionic forms of these amino acids either adsorb through their side chains (lysine, alanine, serine, threonine) or are not directly adsorbed to the surface (glutamic acid and aspartic acid) and have the greatest energy minima at ∼0.6 nm separation, indicating the presence of a water layer between the amino acids and graphene. In the case of the capped anionic amino acids (lysine and the polar amino acids), this is likely due to the preferential solvation of the charged side chains. In their zwitterionic forms, the charged termini of lysine, serine, threonine, and alanine have a greater charge density than the side chains, and so the termini are oriented toward the solvent. Zwitterionic glutamic acid and aspartic acid are both highly charged anionic molecules, and so direct contact with the graphene surface (requiring desolvation of parts of the amino acids) is unfavorable. Finally, capped alanine likely adsorbs through its backbone because its side chain is much smaller than the backbone and so adsorption through the backbone reduces water contact with the relatively hydrophobic amino acid.

The strongest absorbing amino acids, in both their capped and zwitterionic forms, were the aromatic amino acids tryptophan (Trp), tyrosine (Tyr), and phenylalanine (Phe) and the cationic amino acid arginine (Arg). All four of these amino acids adsorbed to the surface through their side chains (Figure 4 and Supporting Information Figures S12 and S13). This matches the trends observed in other studies, where the aromatic and cationic amino acids (particularly arginine) bound most strongly due to pi-pi and cation-pi interactions.^39,40^ While experimental data are limited, available data suggest that the aromatic amino acids and arginine bind most strongly to graphene and carbon nanotubes.^54,56,81^ This means that the application of PyC materials such as biochar to soils could reduce the availability of these amino acids. Both arginine and tryptophan are known to promote plant growth and aid in stress response, and a reduction in the availability of these amino acids may affect plant growth and yield.^22–24,26–28^

### 3.3 Effect of Ionic Strength

Ionic strength had, on average, an insignificant effect on the adsorption free energies of amino acids (Figure 5). The average difference for the zwitterionic amino acids was 0.6 ± 1.4 kJ/mol (*P*-value: 0.838) and for the capped amino acids it was 0.5 ± 2.6 kJ/mol (*P*-value: 0.796). The effect of ionic strength, however, appears significant for specific amino acids, based on statistical tests. However, the base thermal noise (RT, where *R* = 8.314 J/(mol·K)) is around 2.5 kJ/mol at 300 K, and many of the differences in free energies of adsorption at the different ionic strengths are below this value and are therefore considered not significant. The capped amino acids with a significant difference (*P*-values < 0.05) above the thermal noise in the energies of adsorption are aspartic acid (Asp), arginine (Arg), and tryptophan (Trp) (Figure 5 and Supporting Information Tables S2, S3, and S4). The differences in free energies of adsorption for most zwitterionic amino acids are insignificant (Figure 5 and Supporting Information Table S4). The reasons for these differences in adsorption energies for the capped amino acids are not clear and should be explored further.

**Figure 5:**
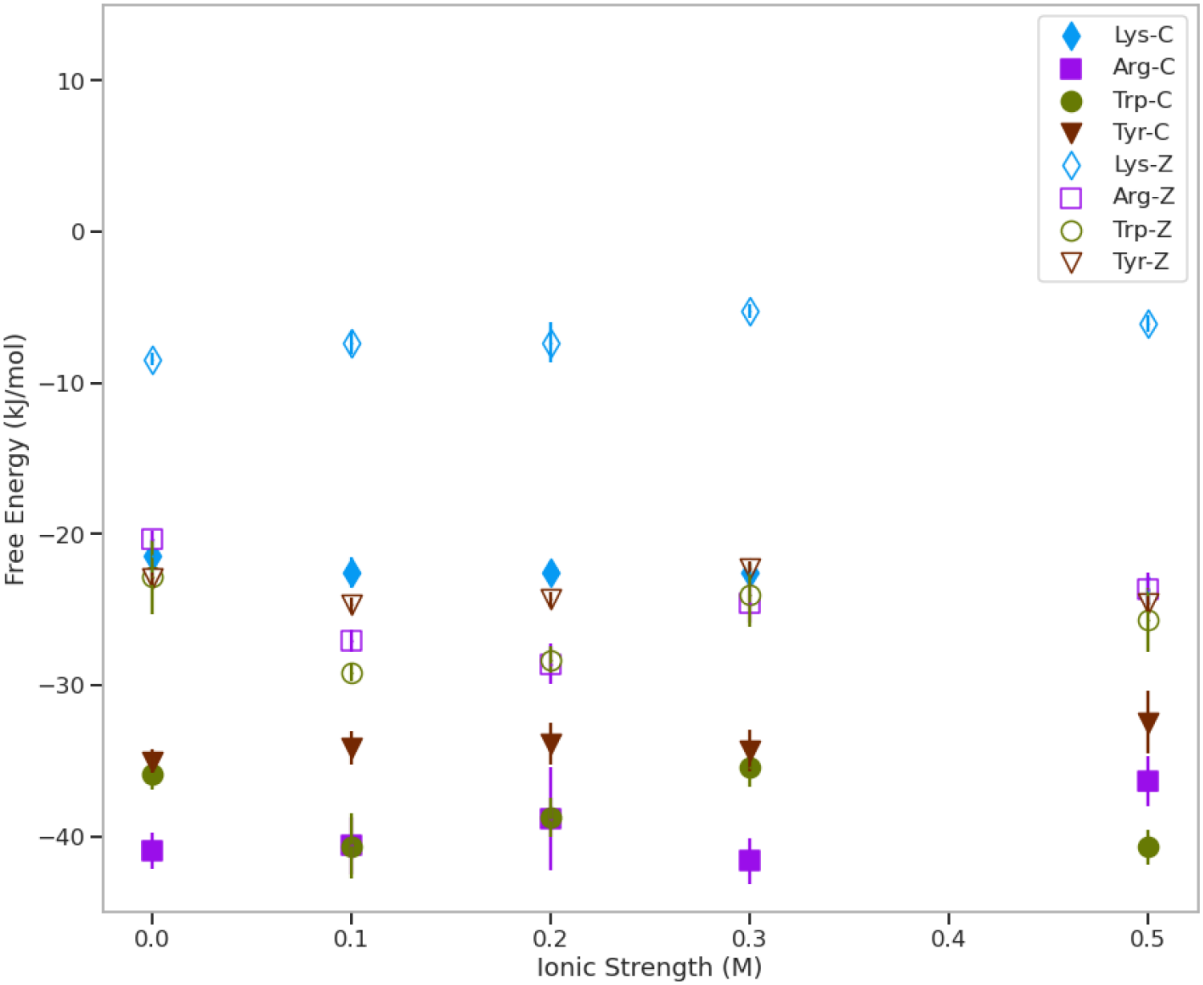
Free energies of adsorption of Lys (blue diamonds), Arg (purple squares), Trp (green circles), and Tyr (brown triangles), in their capped (filled) and zwitterionic (unfilled) forms at ionic strengths of 0, 0.1, 0.3, and 0.5 M NaCl.

To further explore the possible effects of ionic strength, the adsorption free energies of two of the aromatic amino acids (tryptophan and tyrosine) and the cationic amino acids (arginine and lysine) were tested at 0.1, 0.2, and 0.3 M NaCl (Figure 5 and Supporting Information Table S3). While there are differences in the adsorption free energies at different ionic strengths for some amino acids, there are no clear trends in the adsorption free energies across the range of ionic strengths tested (0–0.5 M NaCl). This suggests that varying the ionic strength may not have a large impact on amino acid adsorption to graphene in zwitterionic or capped forms. However, while ionic strength may not impact protein adsorption at the individual amino acid level, it may impact protein adsorption due to the increase in size and reduction in polarity of proteins compared to amino acids, which increase the strength of salting-out effects.^58,59^

## 4 Conclusions

The findings of this study have several important implications for the adsorption of proteins and amino acids in the environment. First, the Interface force field for graphene, which explicitly accounts for graphene’s delocalized electrons, can simulate the interactions between the 20 natural amino acids and graphene that largely align with reported simulation and experimental results. The adsorption free energies calculated using IFF are also close to previous results calculated using nonpolarizable force fields, except for the significantly stronger adsorption of arginine due to stronger cation-π interactions. It is also noted that there are currently no experimental data available to determine whether IFF or traditional force fields are more accurate, which should be assessed in the future once such data become available. Second, tryptophan, tyrosine, arginine, and phenylalanine absorbed most strongly to graphene, indicating that the addition of PyC materials to soils and waters could reduce the bioavailability of these amino acids. Third, the capped amino acids bound more strongly to graphene, which is what we would expect for larger, more neutral molecules. Moreover, some amino acids including lysine, aspartic acid, glutamic acid, serine, threonine, and alanine adsorbed to graphene through the capped peptide bonds, indicating that the peptide backbone may play a role in protein adsorption to graphene, especially when the amino acid side chains do not adsorb strongly to graphene surfaces. In instances where the amino acid side chains do interact strongly with graphene, including the aromatic amino acids and arginine, the amino acids adsorbed to graphene through their side chains. This can provide insight into how proteins might orient when they adsorb to graphene to maximize the favorability of the interactions. Moreover, proteins can change their conformation when they adsorb to surfaces,^82,83^ and so insights into which orientations of amino acids are more favorable for attraction to the surface can help predict conformation changes of proteins on surfaces. Finally, we found that ionic strength had, on average, an insignificant effect on amino acid adsorption, and there were no clear trends in the adsorption free energies of the amino acids over the ionic strength range of 0–0.5 M NaCl. This likely indicates that the adsorption of amino acids to graphene is not sensitive to ionic strength. However, PyC materials may have other surface functional groups, and interactions between these groups and amino acids could be more sensitive to ionic strength. Ionic strength could also have an impact on the adsorption of larger molecules such as proteins due to salting-out effects. Future studies should simulate the interaction of peptides and proteins with graphene in different ionic strength conditions to provide further insights into the interactions of proteins with PyC materials in the environment.

## Supplementary Material



## Notes

Supplemental figures include Figure S1 Tyrosine in zwitterionic and capped forms; Figures S2–S9 showing umbrella sampling window overlaps; Figures S10–S11 showing Potential of Mean Force (PMF) graphs of zwitterionic and capped amino acids; Figures S12–S13 showing density profiles of zwitterionic and capped amino acids; and supplemental tables summarizing the free energies of adsorption for amino acids reported in the literature and this study (PDF)
