# Supplementary material for: Adsorption of Zwitterionic and Capped Amino Acids to Graphene: A Molecular Dynamics Study
Source: ACS Omega. 2026 Jun 5;11(24):35191–200. doi: 10.1021/acsomega.5c13030 (PMC13294876; doi:10.1021/acsomega.5c13030)
Supplement: Supplementary file 1 [file ao5c13030_si_001.pdf]

Supporting Information for

Adsorption of Zwitterionic and Capped Amino Acids to Graphene: A Molecular Dynamics Study

Antryg Benedict[†], Hui Li[†], Qi Yuan[‡], Jason Bartz[‡], Wei Zhang[†,§,*]

[†]Department of Plant, Soil and Microbial Sciences, Michigan State University, East Lansing, Michigan, 48824, United States

[‡]School of Medicine, Medical Microbiology, and Immunology, Creighton University, Omaha, Nebraska, 68178, United States

[§]Environmental Science and Policy Program, Michigan State University, East Lansing, Michigan 48824, United States

*Corresponding author: Dr. Wei Zhang, Address: 1066 Bogue ST RM A516, East Lansing, MI 48824, United States; Tel: 517-353-0471; Fax: 517-355-0270; Email: weizhang@msu.edu

Submitted to ACS Omega

Content

Figure S1. Tyrosine in its zwitterionic form (top) and capped form (bottom).

Figure S2. Histograms showing umbrella sampling window overlap for zwitterionic amino acids in 0M NaCl.

Figure S3. Close up of near-graphene windows for umbrella sampling histograms of zwitterionic amino acids in 0 M NaCl.

Figure S4. Histograms showing umbrella sampling window overlap for zwitterionic amino acids in 0.5 M NaCl.

Figure S5. Close up of near-graphene windows for umbrella sampling histograms of zwitterionic amino acids in 0.5 M NaCl.

Figure S6. Histograms showing umbrella sampling window overlap for capped amino acids in 0 M NaCl.

Figure S7. Close up of near-graphene windows for umbrella sampling histograms of capped amino acids in 0 M NaCl.

Figure S8. Histograms showing umbrella sampling window overlap for capped amino acids in 0.5 M NaCl

Figure S9. Close up of near-graphene windows for umbrella sampling histograms of capped amino acids in 0.5 M NaCl

Figure S10. Potential of Mean Force (PMF) graphs of zwitterionic amino acids.

Figure S11. Potential of Mean Force (PMF) graphs of capped amino acids

Figure S12. Density profiles: probability (g(r)) of finding moieties of the uncapped amino acids at a given distance (r) from the graphene.

Figure S13. Density profiles: probability (g(r)) of finding moieties of the capped amino acids at a given distance (r) from the graphene.

Table S1 Literature values for free energies of amino acids adsorption to graphene and related materials.

Table S2 Free energies of amino acids adsorption to graphene in 0 M and 0.5 M NaCl.

Table S3 Free energies of adsorption of amino acids at different ionic strengths.

Table S4 Difference between free energies of adsorption of zwitterionic and capped amino acids to graphene at different ionic strengths.

17 pages, 13 figures, and 3 Tables

49

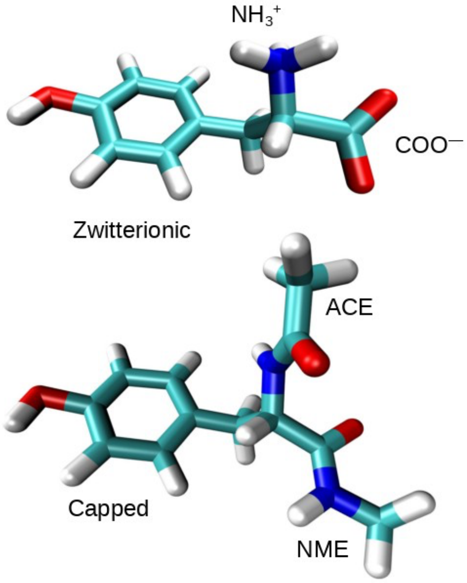

50 **Figure S1.** Tyrosine in its zwitterionic form (top) and capped form (bottom).

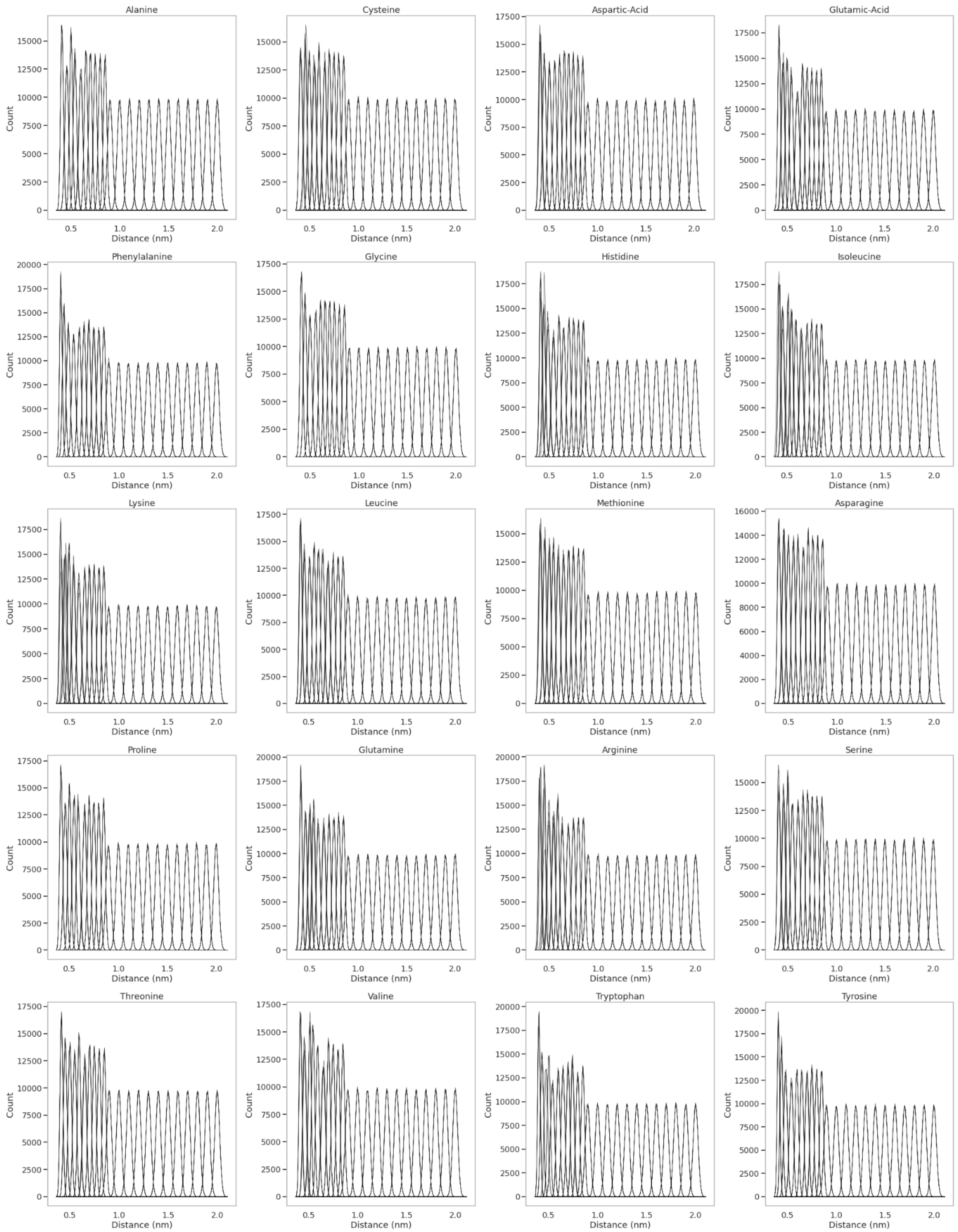

Figure S2. Histograms showing umbrella sampling window overlap for zwitterionic amino acids in 0M NaCl.

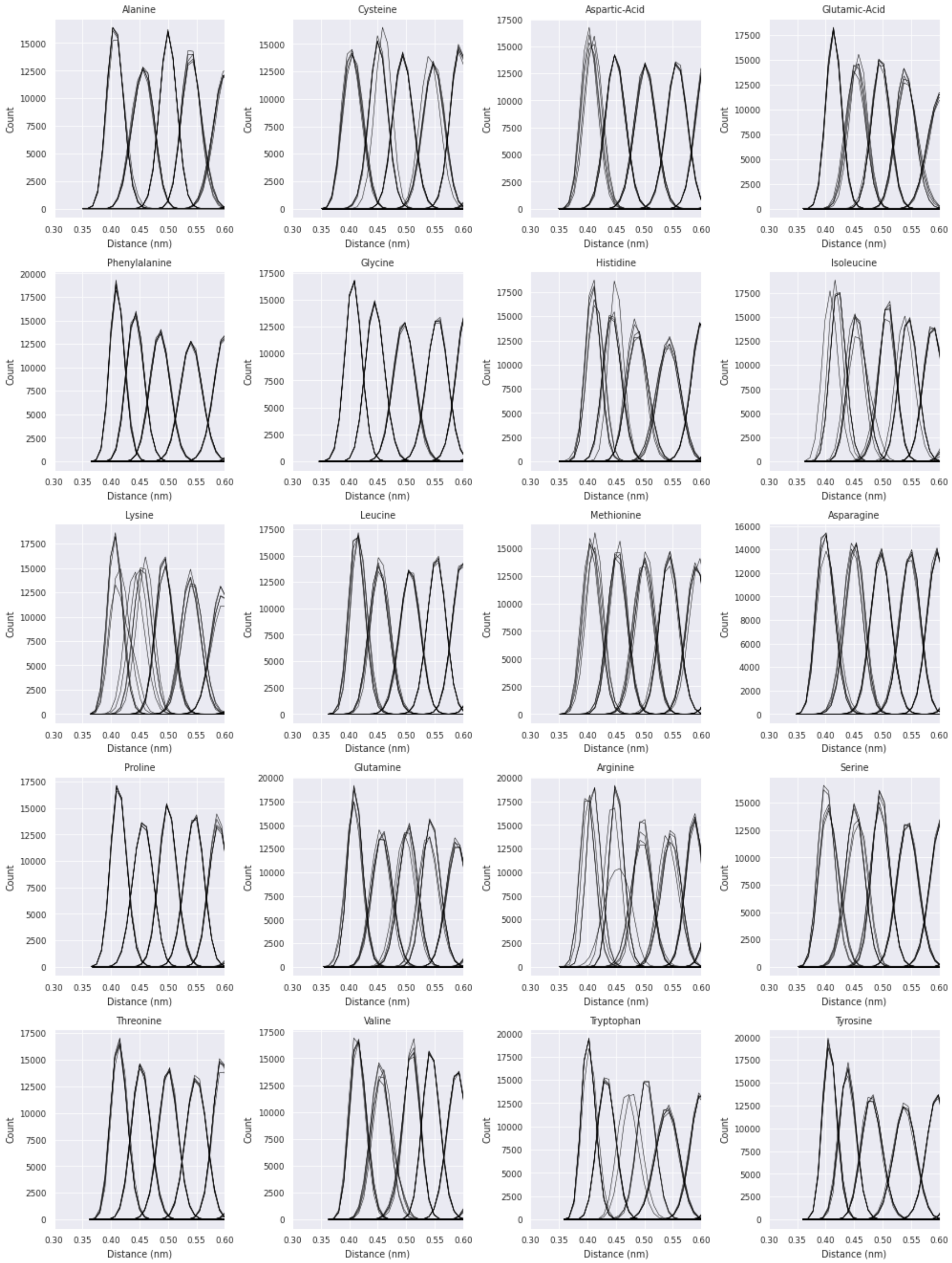

Figure S3. Close up of near-graphene windows for umbrella sampling histograms of zwitterionic amino acids in 0 M NaCl.

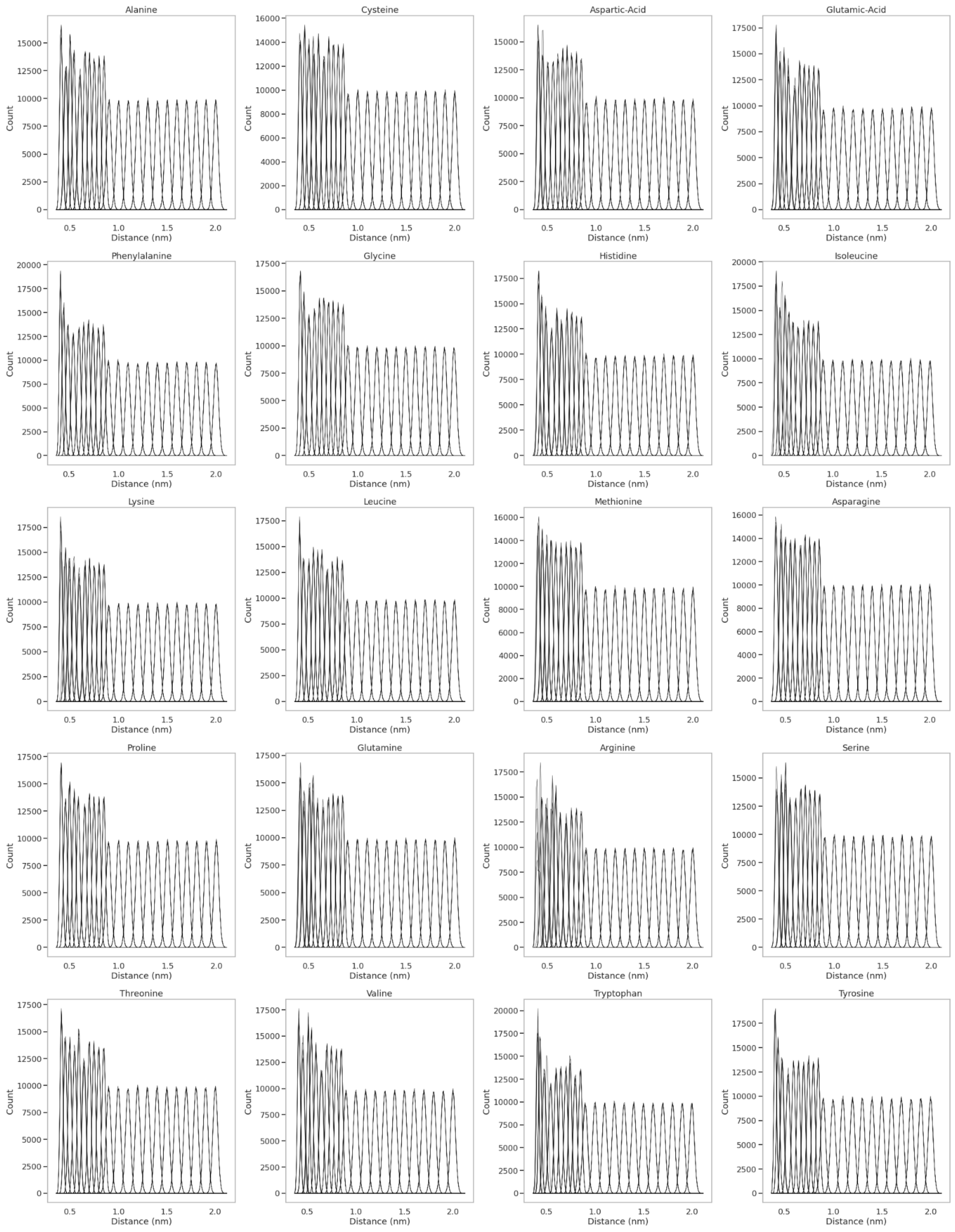

Figure S4. Histograms showing umbrella sampling window overlap for zwitterionic amino acids in 0.5 M NaCl.

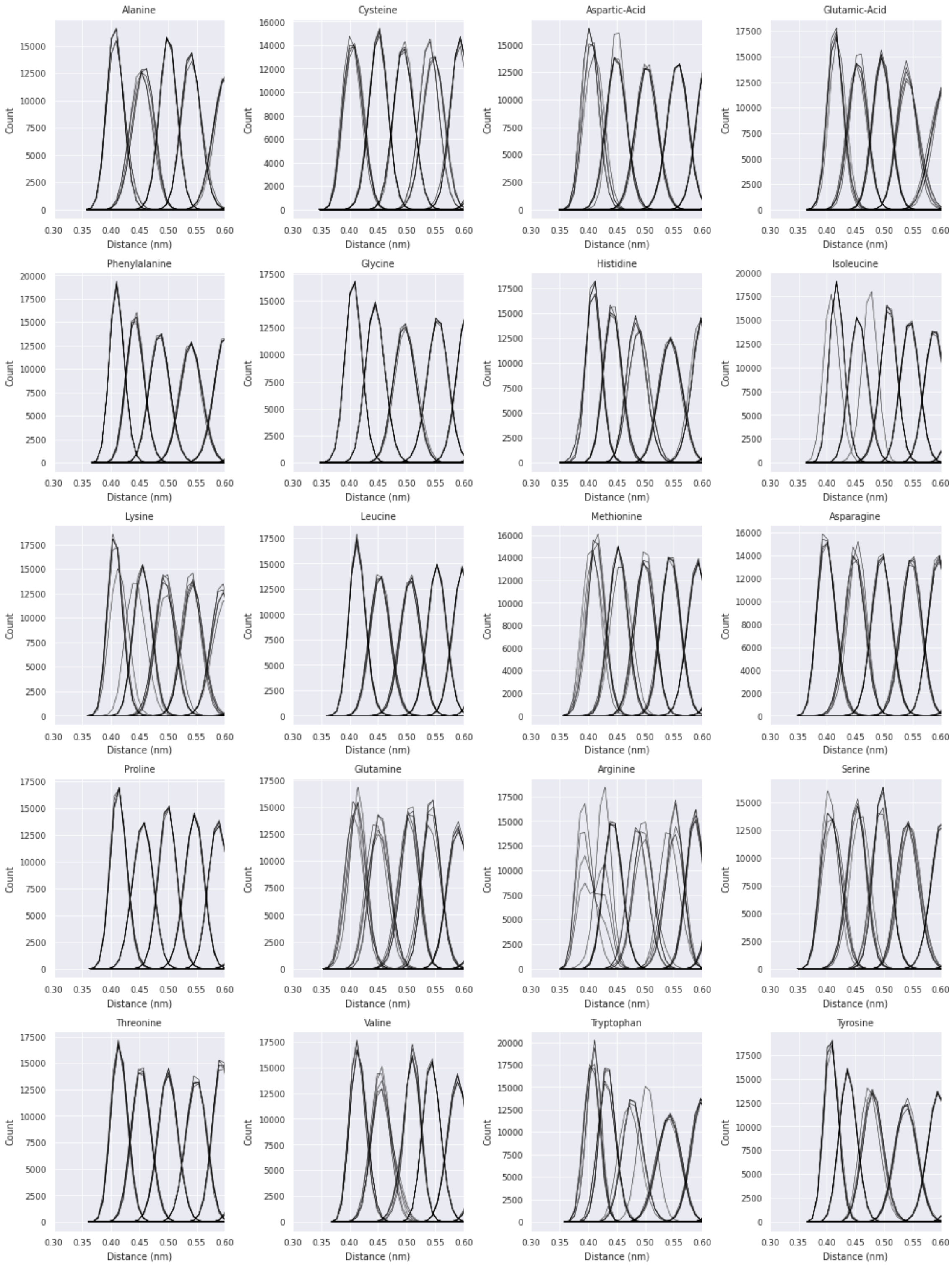

Figure S5. Close up of near-graphene windows for umbrella sampling histograms of zwitterionic amino acids in 0.5 M NaCl.

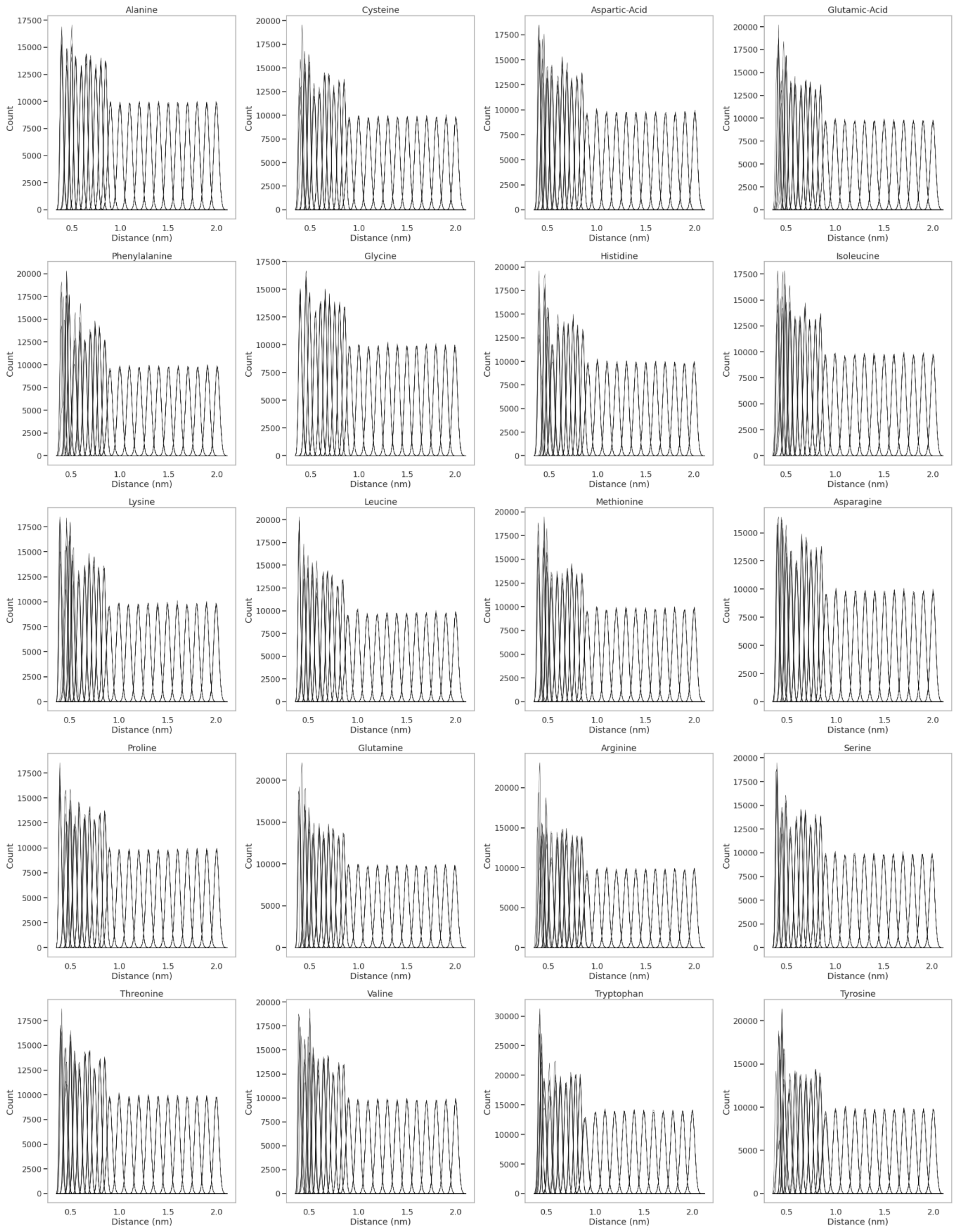

Figure S6. Histograms showing umbrella sampling window overlap for capped amino acids in 0 M NaCl.

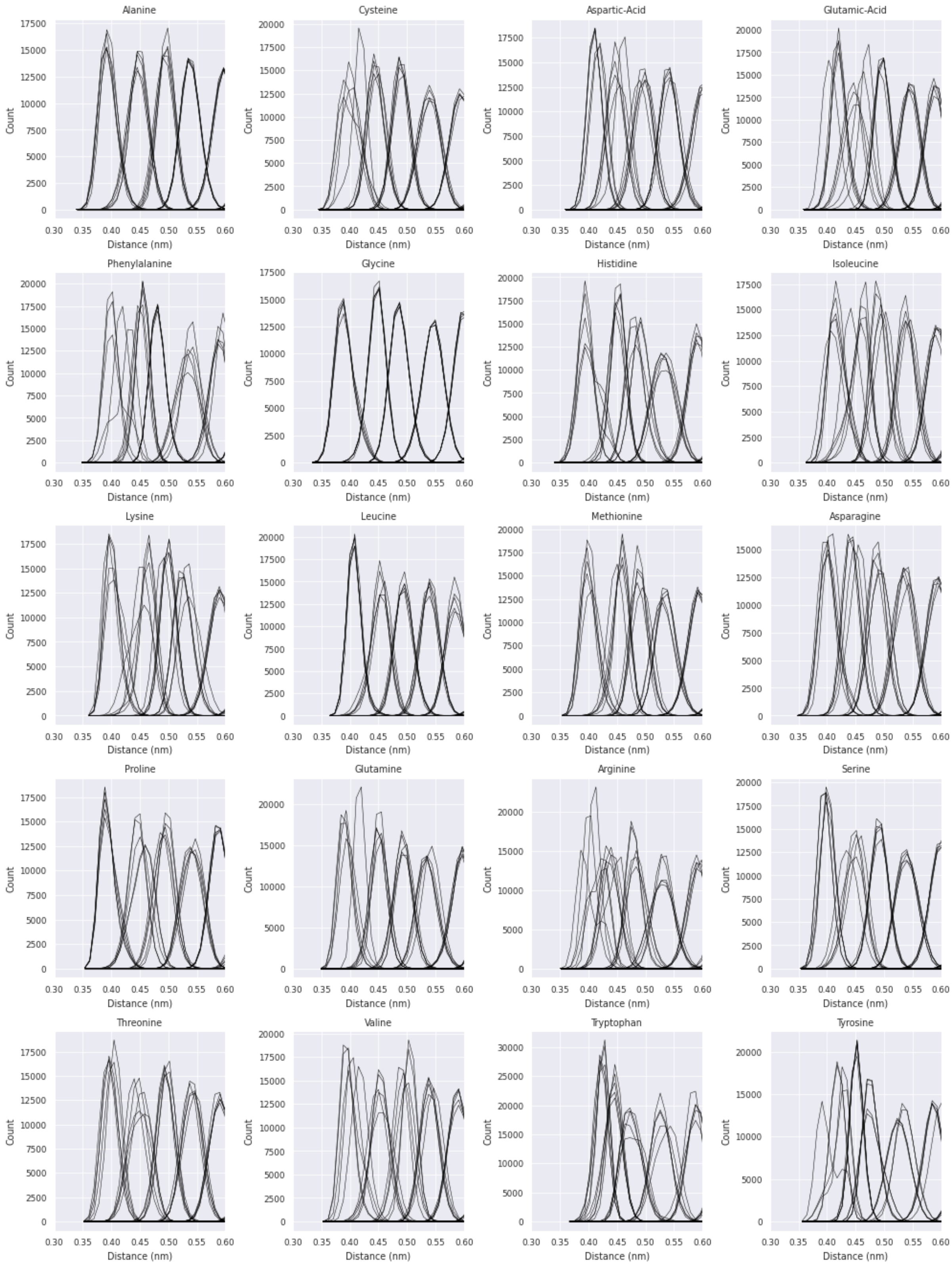

Figure S7. Close up of near-graphene windows for umbrella sampling histograms of capped amino acids in 0 M NaCl.

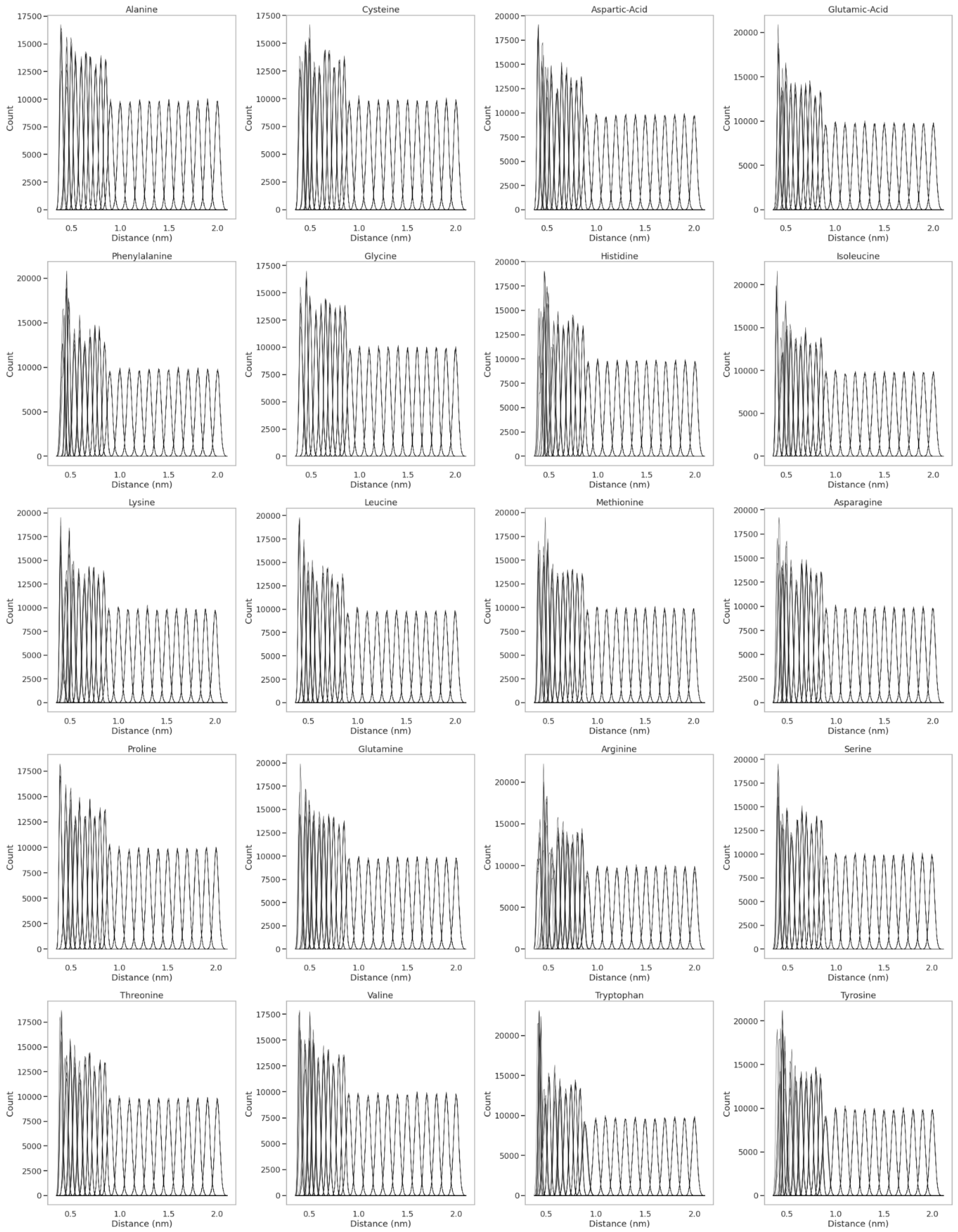

Figure S8. Histograms showing umbrella sampling window overlap for capped amino acids in 0.5 M NaCl.

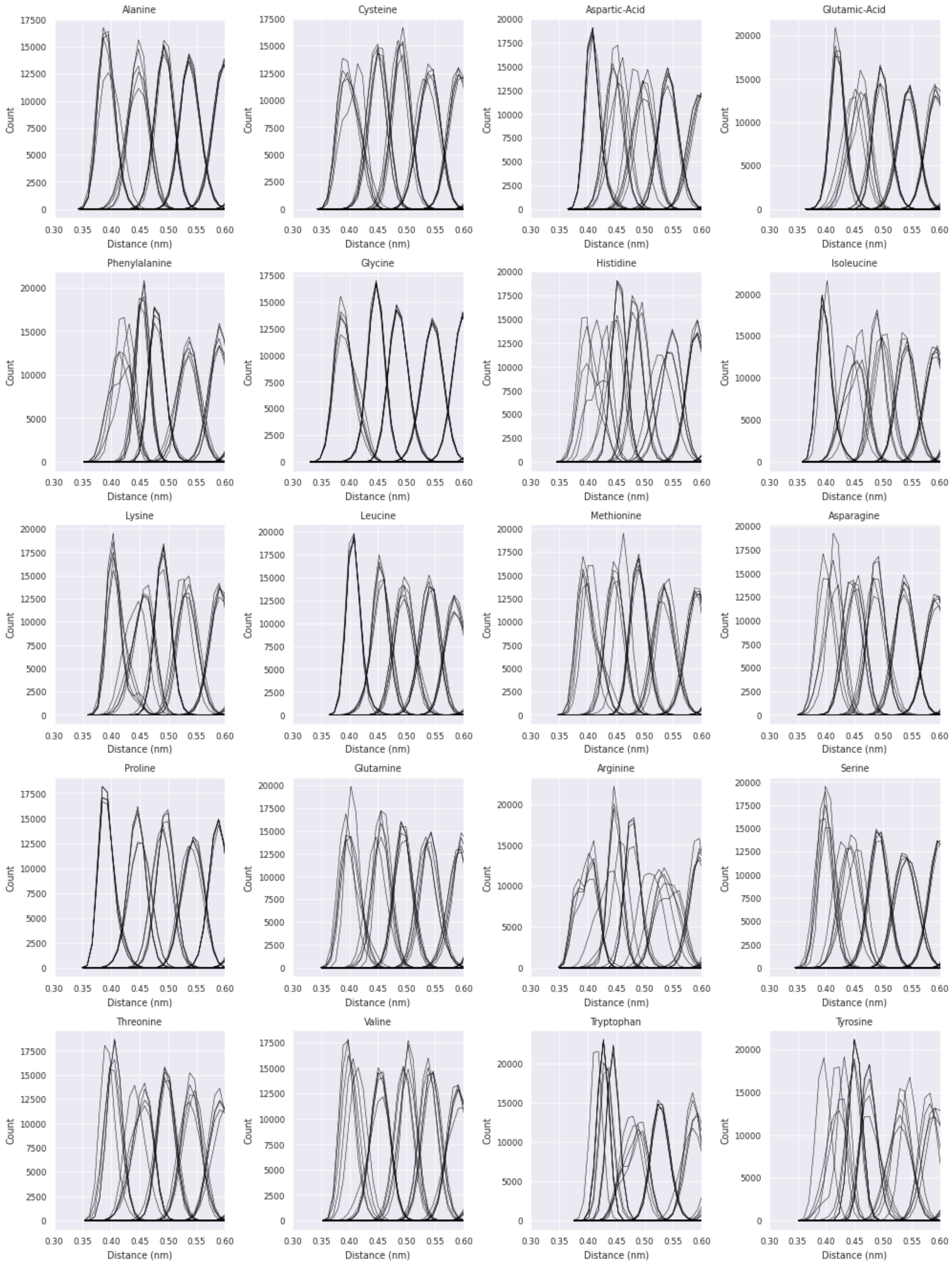

Figure S9. Close up of near-graphene windows for umbrella sampling histograms of capped amino acids in 0.5 M NaCl.

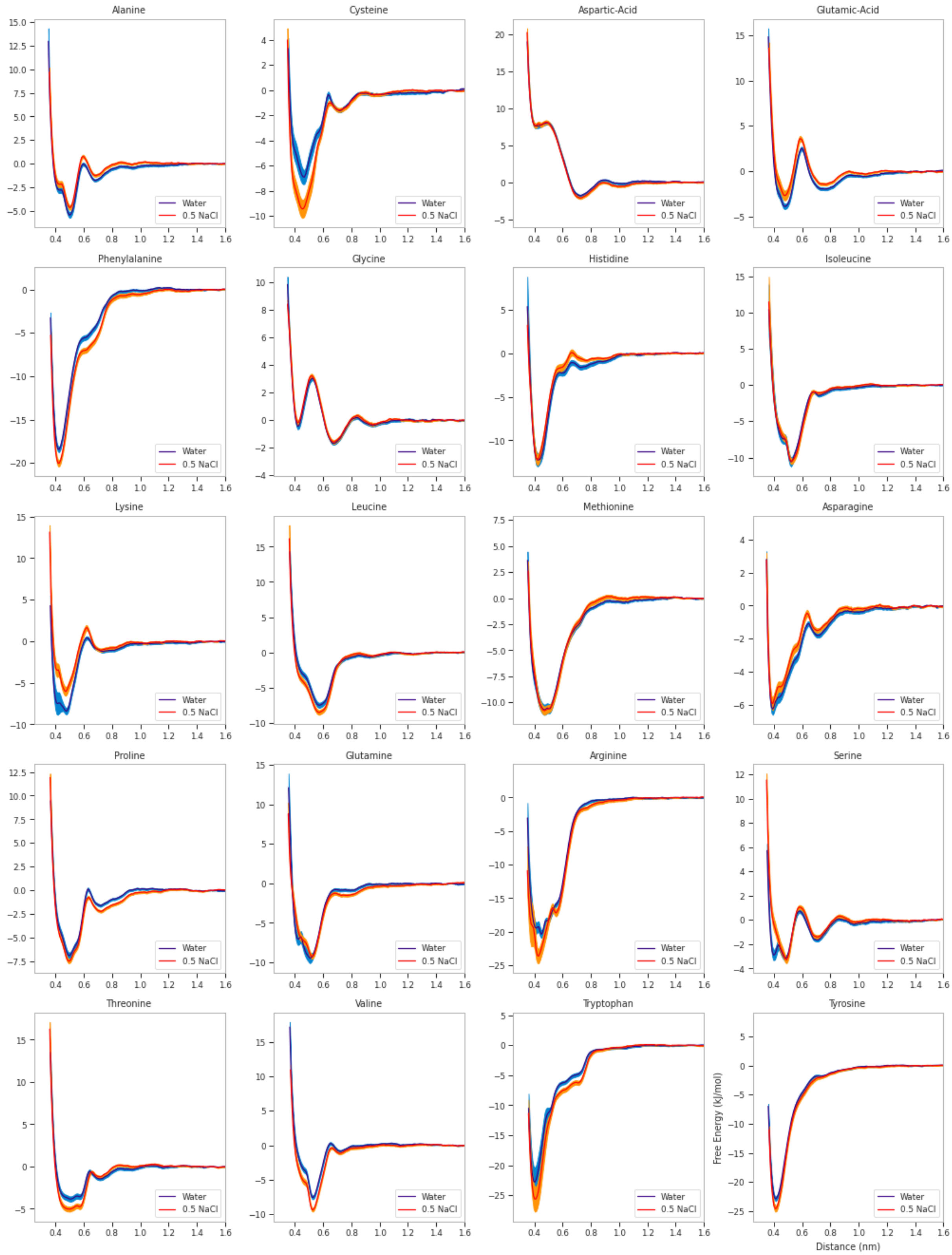

Figure S10. Potential of Mean Force (PMF) graphs of uncapped amino acids.

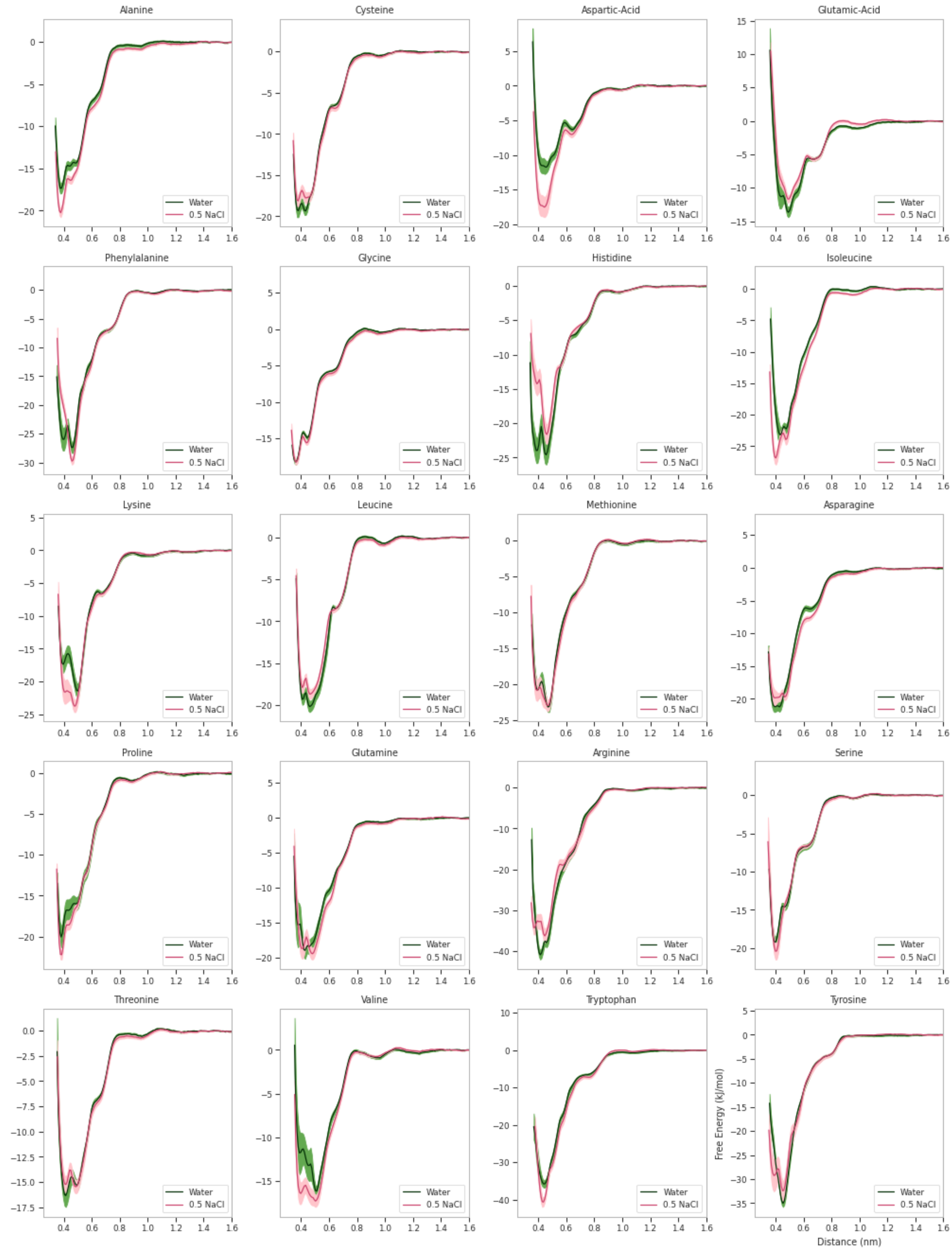

Figure S11. Potential of Mean Force (PMF) graphs of capped amino acids.

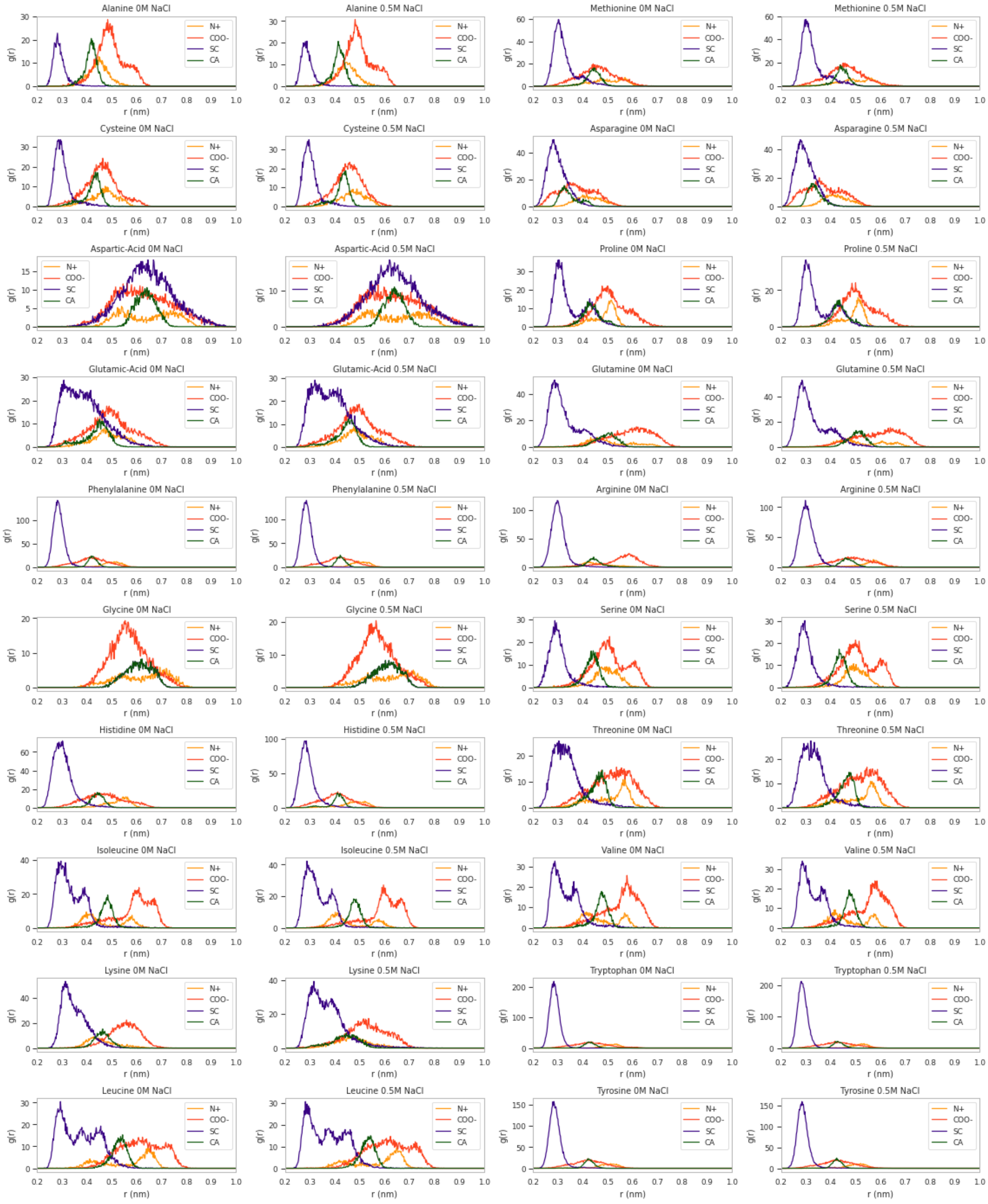

Figure S12. Density profiles: probability (g(r)) of finding moieties of the uncapped amino acids at a given distance (r) from the graphene. N+ and COO- are the termini, SC is the side chain, and CA is the alpha carbon.

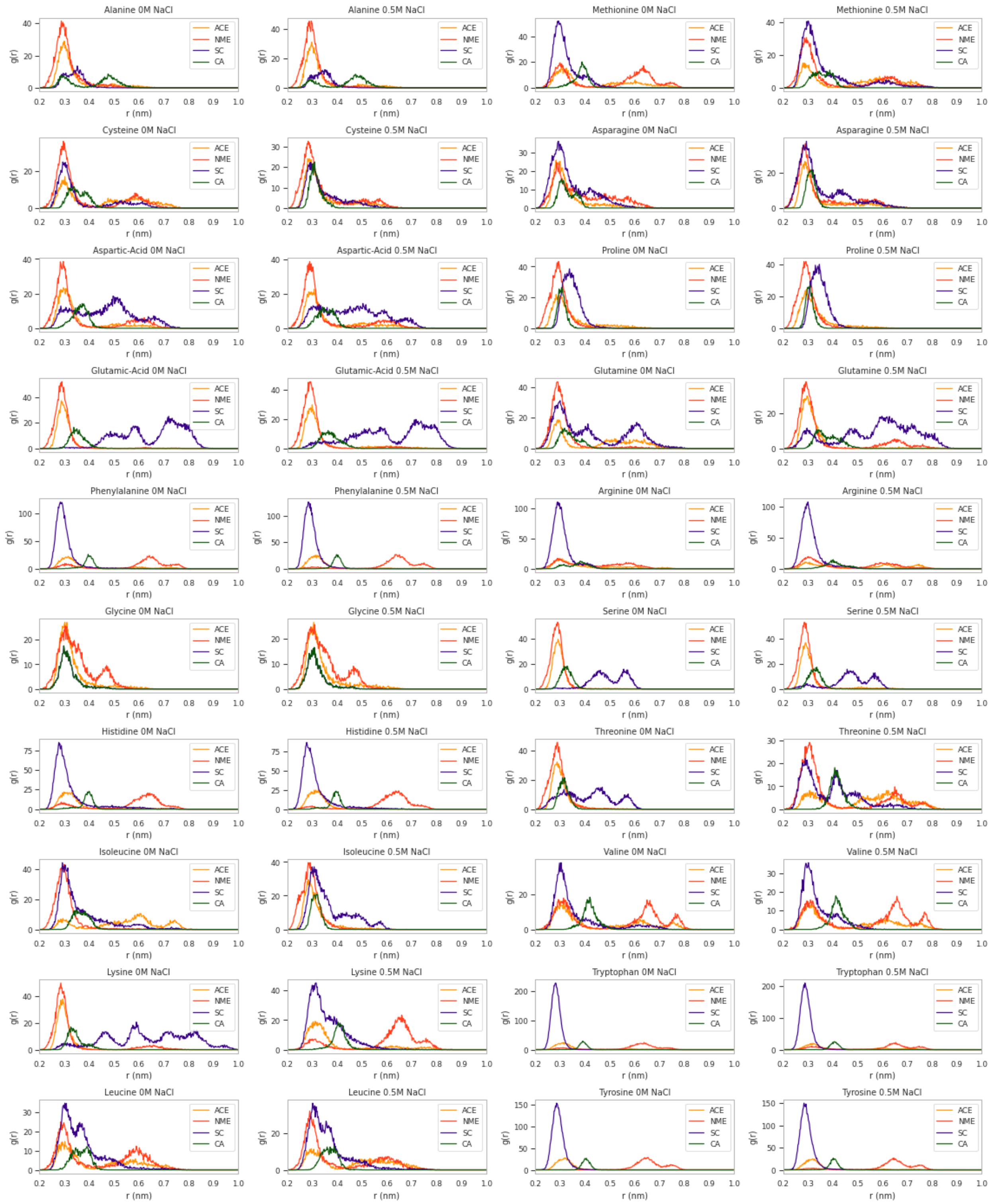

Figure S13. Density profiles: probability (g(r)) of finding moieties of the capped amino acids at a given distance (r) from the graphene. ACE and NME are the termini, SC is the side chain, and CA is the alpha carbon.

Table S1 Literature values for free energies of amino acids adsorption to graphene and related materials.

Amino Acids																				Surface	Method	Reference
Ala	Arg	Asn	Asp	Cys	Gln	Glu	Gly	His	Ile	Leu	Lys	Met	Phe	Pro	Ser	Thr	Tyr	Trp	Val			
Energies of Adsorption (kJ/mol)																						
-9.6 ± 0.1	-18.5 ± 0.0	-13.9 ± 0.0	-9.5 ± 0.0	-13.3 ± 0.0	-16.4 ± 0.0	-7.5 ± 0.0	-8.0 ± 0.0	-18.2 ± 0.0	-14.3 ± 0.1	-15.3 ± 0.0	-11.4 ± 0.1	-19.4 ± 0.0	-18.4 ± 0.1	-6.0 ± 0.0	-11.2 ± 0.0	-13.0 ± 0.0	-27.5 ± 0.0	-26.2 ± 0.0	-12.6 ± 0.0	Graphene	Molecular Dynamics, GROMOS 54A8 FF, neutral termini, ΔA	Barria-Urenda et al, 2023
-12.1 ± 3.3	-43.5 ± 1.5	-34.3 ± 0.8	-19.7 ± 3.3	-16.7 ± 1.3	-36.8 ± 0.8	-23.4 ± 5.4	-31.8 ± 3.8	-26.4 ± 2.9	-12.1 ± 5.9	-13.0 ± 3.3	-32.6 ± 3.3	-22.6 ± 1.7	-13.4 ± 2.9	-18.8 ± 1.7	-19.2 ± 4.6	-15.5 ± 1.3	-26.4 ± 2.9	-29.7 ± 2.1	-4.2 ± 2.9	Graphene	Molecular Dynamics,TEAMS FF, GXG peptides, ΔH	Camden et al, 2013
-28.0	-39.7	-47.3	-31.4	-11.3	-38.5	-25.1	-33.1	-26.8	-5.4	-5.9	-32.6	-25.1	-8.8	-7.1	-21.8	-15.9	-31.4	-26.4	-13.0		Molecular Dynamics, TEAMS FF, GXG peptides, ΔH	Welch et al, 2015
3.7	-11.7	-8.9	0.0	-2.0	-10.8	-3.1	16.3	-16.5	-1.1	6.6	-5.0	-5.8	-11.7	-4.0	-2.9	-1.1	8.7	-21.2	-0.2	Graphene	Molecular Dynamics, Amber03, Zwitterionic, ΔH	Dragneva et al, 2013
-32.6	-69.9	-32.3	-29.5	-27.7	-35.4	-33.9	-34.6	-36.3	-29.3	-27.5	-32.2	-34.0	-29.9	-23.1	-31.7	-23.3	-38.5	-43.4	-29.6	Graphene	Molecular Dynamics, Amber03, Capped, ΔH	Dragneva et al, 2013
-10.4 ± 1.3	-23.2 ± 0.7	-15.3 ± 2.7	-8.4 ± 1.1	-10.8 ± 0.7	-21.8 ± 0.9	-11.0 ± 0.9	-18.7 ± 1.6	-13.4 ± 1.5	-2.8 ± 0.8	-7.2 ± 0.9	-6.9 ± 0.7	-16.5 ± 2.1	-14.9 ± 1.5	-7.2 ± 0.7	-13.3 ± 2.1	-10.5 ± 1.0	-20.8 ± 1.1	-21.4 ± 2.9	-7.6 ± 1.1	Graphene	Molecular Dynamics, GRAPPA, Capped, ΔA	Hughes and Walsh, 2015
-10.4 ± 0.3	-30.5 ± 0.4	-15.6 ± 0.5	-8.8 ± 0.9	-15.1 ± 0.6	-17.8± 0.4	-10.8 ± 0.5	-9.6 ± 0.2	-22.1 ± 0.3	-15.2 ± 1.0	-13.2 ± 0.4	-12.6 ± 1.0	-18.4 ± 1.3	-25.6 ± 0.9	-12.8 ± 0.4	-13.5 ± 0.3	-11.8 ± 0.8	-33.4 ± 0.5	-37.6 ± 0.7	-14.1 ± 0.6	Graphene	Molecular Dynamics, Amber99SB and GAFF, Zwitterionic, ΔA	Hirano and Kameda, 2021
-22.5 ± 1.1	-46.0 ± 1.7	-25.0 ± 0.8	-21.2 ± 0.8	-23.5 ± 0.7	-28.3 ± 1.0	-21.7 ± 0.7	-28.3 ± 0.4	-32.1 ± 0.8	-22.6 ± 1.1	-23.5 ± 0.7	-26.8 ± 1.1	-28.4 ± 0.8	-35.2 ± 0.8	-25.0 ± 0.7	-23.8 ± 0.8	-20.0 ± 0.8	-45.3 ± 1.2	-46.5 ± 1.6	-22.0 ± 1.1	Graphene	Molecular Dynamics, Amberff99SB-ILDN, capped, ΔA	Dasetty et al, 2019
-16.5 ± 0.5	-31.2 ± 2.4	-21.5 ± 1.1	-12.5 ± 0.7	-20.3 ± 0.9	-20.3 ± 1.0	-14.9 ± 0.6	-17.6 ± 0.3	-21.6 ± 1.1	-18.5 ± 1.0	-20.0 ± 0.8	-20.0 ± 1.0	-25.0 ± 0.8	-31.2 ± 1.0	-21.4 ± 0.7	-18.0 ± 0.8	-19.3 ± 0.8	-33.8 ± 1.2	-39.6 ± 1.7	-16.1 ± 0.8	Graphene	Molecular Dynamics, CHARMM36, capped, ΔA	Dasetty et al, 2019
-25.1 ± 0.6	-41.9 ± 1.8	-27.4 ± 0.8	-22.5 ± 0.8	-26.2 ± 0.7	-30.6 ± 1.7	-24.2 ± 0.9	-30.6 ± 0.4	-31.8 ± 0.8	-22.4 ± 0.6	-25.6 ± 0.5	-29.7 ± 1.7	-30.4 ± 0.5	-34.4 ± 0.6	-28.2 ± 0.7	-26.2 ± 0.7	-22.8 ± 0.7	-41.3 ± 1.0	-48.8 ± 1.2	-20.9 ± 0.6	Graphene	Molecular Dynamics, OPLS-AA/M, capped, ΔA	Dasetty et al, 2019
-22.5 ± 1.4	-43.1 ± 2.5	-29.4 ± 1.6	-20.7 ± 1.0	-25.1 ± 0.6	-31.4 ± 1.8	-22.4 ± 1.0	-32.0 ± 0.5	-37.5 ± 1.2	-29.0 ± 1.7	-28.6 ± 1.2	-29.6 ± 1.4	-36.0 ± 1.3	-40.7 ± 1.1	-24.4 ± 1.3	-24.9 ± 0.7	-23.2 ± 1.0	-41.2 ± 1.6	-54.0 ± 1.9	-28.6 ± 1.1	Graphene	Molecular Dynamics, Amber03w, capped, ΔA	Dasetty et al, 2019
-37.7	-84.1	-56.9	-46.9	-46.9	-67.4	-63.2	-32.2	-66.5	-55.6	-56.5	-69.0	-64.4	-77.0	-48.1	-43.5	50.2	-79.1	-98.7	-49.0	Graphene	Molecular Dynamics, Amberff99SB, capped, ΔH	Pandey et al, 2012
-21.0	-76.8	-20.7	-67.2	-21.4	-21.0	-67.8	-24.3	-23.7	-22.2	-23.1	-24.3	-22.6	-23.7	-19.3	-18.0	-20.3	-31.6	-34.9	-20.1	Graphene	Molecular Dynamics, CHARMM Drude Polarizable, capped, ΔG	Soni et al, 2025
-18.3	-42.6	-23.4	-16.5	-22.9	-21.2	-18.3	-27.9	-26.0	-18.3	-21.2	-21.3	-26.2	-31.4	-24.6	-19.2	-21.3	-36.9	-42.1	-18.3	Graphene	Molecular Dynamics, CHARMM36m and CGENFF, capped, ΔG	Soni et al, 2025
-14.2	-45.1	-21.9	-18.9	-21.7	-26.6	-21.7	-13.8	-30.6	-21.6	-19.4	-31.9	-25.9	-35.3	-18.9	-18.0	-17.6	-41.1	-49.6	-18.6	Carbon Nanotube	Molecular Dynamics, CHARMM27, zwitterionic, interaction energy	He and Zhou, 2014
-1.23	-17.20	-7.21	-2.32	-3.51	-8.47	-3.96	-8.56	-13.95	-8.60	-7.14	-5.91	-9.14	-15.64	-14.04	-2.72	-4.38	-21.38	-24.29	-5.56	Graphene	Molecular Dynamics, GAFF, side chain analogues, ΔA	Saeedimasine et al, 2020
-0.08	-11.16	-5.09	-0.20	-1.54	-5.64	-2.26	-5.39	-9.64	-5.18	-4.39	-3.64	-5.60	-8.88	-10.12	-1.10	-2.56	-13.35	-13.51	-3.94	Carbon Nanotube	Molecular Dynamics, GAFF, side chain analogues, ΔA	Saeedimasine et al, 2020
Fraction Adsorbed																						
	0.65	0.12	0.06	0.56	0.16	0.08		0.55	0.3	0.32	0.08	0.62	0.91		0.06	0.05	0.85		0.1	SWNT	Adsorption Study	Guo et al, 2008
Retention Time (minute)																						
1.80 ± 0.01							1.81 ± 0.01	2.1 ± 0.02	1.86 ± 0.02	1.85 ± 0.01			2.07 ± 0.02				2.11 ± 0.02	4.44 ± 0.23	1.83 ± 0.01	SWNT-NH_2-silica	Liquid chromatography	Iwashita et al, 2015
Energies of Adsorption (kJ/mol)																						
ND	-19.24	-13.91	ND			ND	ND	-20.06		ND	-17.42		-18.73		-14.99		-19.16	-20.87		Graphene Oxide	Isothermal Calorimetry	Pandit and De, 2016

Table S2 Free energies of amino acids adsorption to graphene in 0 M and 0.5 M NaCl.

Amino Acids	Zwitterion 0M (kJ/mol)	Error Zwitterion 0M (kJ/mol)	Zwitterion 0.5M (kJ/mol)	Error Zwitterion 0.5M (kJ/mol)	Capped 0M (kJ/mol)	Error Capped 0M (kJ/mol)	Capped 0.5M (kJ/mol)	Error Capped 0.5M (kJ/mol)
Ala	-5.46	0.32	-4.64	0.26	-17.33	0.62	-20.24	0.55
Cys	-6.90	0.56	-9.44	0.72	-19.34	0.51	-18.13	0.71
Asp	-2.25	0.17	-2.13	0.17	-11.75	0.89	-17.52	1.46
Glu	-3.91	0.31	-2.70	0.55	-13.66	0.75	-11.69	0.52
Phe	-18.44	0.33	-20.03	0.39	-27.38	0.96	-29.75	0.63
Gly	-1.71	0.14	-1.74	0.14	-18.23	0.38	-18.22	0.39
His	-12.24	0.67	-12.22	0.63	-24.60	1.44	-21.64	1.41
Ile	-10.61	0.63	-10.53	0.41	-23.19	1.30	-26.85	1.07
Lys	-8.42	0.41	-6.04	0.53	-21.44	1.10	-23.74	0.95
Leu	-7.53	0.37	-8.56	0.35	-20.14	0.67	-18.65	0.59
Met	-10.70	0.50	-10.80	0.41	-23.19	0.72	-23.01	0.79
Asn	-6.27	0.36	-5.95	0.39	-21.23	0.76	-19.86	1.21
Pro	-6.91	0.33	-7.49	0.23	-20.04	1.30	-22.20	0.60
Gln	-9.45	0.67	-9.26	0.30	-18.98	1.20	-19.45	0.84
Arg	-20.36	0.59	-23.61	1.08	-40.92	1.17	-36.29	1.65
Ser	-3.15	0.22	-3.29	0.30	-19.23	0.70	-20.41	1.09
Thr	-3.85	0.33	-5.05	0.31	-16.35	1.14	-15.48	0.67
Val	-7.57	0.30	-9.40	0.27	-16.18	0.57	-17.33	0.69
Trp	-22.82	2.43	-25.65	2.10	-35.82	1.02	-40.69	1.12
Tyr	-22.91	0.41	-24.58	0.53	-35.03	0.79	-32.42	2.09

Table S3 Free energies of adsorption of amino acids at different ionic strengths.

Amino Acids	0 M (kJ/mol)	0M Error (kJ/mol)	0.10 M (kJ/mol)	0.1 M Error (kJ/mol)	0.20 M (kJ/mol)	0.2 M Error (kJ/mol)	0.30 M (kJ/mol)	0.3 M Error (kJ/mol)	0.50 M (kJ/mol)	0.5 M Error (kJ/mol)
Lys-C	-21.44	1.10	-22.52	1.03	-22.53	0.74	-22.51	0.57	-23.74	0.95
Arg-C	-40.92	1.17	-40.59	1.86	-38.79	3.39	-41.60	1.51	-36.29	1.65
Trp-C	-35.82	1.02	-40.64	2.16	-38.73	1.25	-35.40	1.31	-40.69	1.12
Tyr-C	-35.03	0.79	-34.12	1.10	-33.84	1.36	-34.32	1.36	-32.42	2.09
Lys-Z	-8.42	0.41	-7.32	0.76	-7.33	1.34	-5.25	0.46	-6.04	0.53
Arg-Z	-20.36	0.59	-27.05	0.72	-28.56	1.32	-24.52	1.36	-23.61	1.08
Trp-Z	-22.82	2.43	-29.12	0.58	-28.29	0.87	-24.04	2.12	-25.65	2.10
Tyr-Z	-22.91	0.41	-24.69	0.54	-24.29	0.51	-22.22	0.39	-24.58	0.53

Table S4 Difference between free energies of adsorption of zwitterionic and capped amino acids to graphene at different ionic strengths.

AA	Z Diff (kJ/mol)	Z Diff Error (kJ/mol)	Z Pvals	C Diff (kJ/mol)	C Diff Error (kJ/mol)	C Pvals
Ala	0.82	0.42	2.30E-03	2.91	0.82	1.00E-04
Cys	2.54	0.92	3.00E-04	1.21	0.88	1.48E-02
Asp	0.12	0.24	2.73E-01	5.78	1.71	1.00E-04
Glu	1.21	0.63	2.70E-03	1.97	0.92	1.40E-03
Phe	1.60	0.51	1.00E-04	2.37	1.15	1.70E-03
Gly	0.03	0.20	7.26E-01	0.01	0.55	9.76E-01
His	0.02	0.92	9.64E-01	2.96	2.02	1.11E-02
Ile	0.08	0.76	8.09E-01	3.67	1.68	1.20E-03
Lys	2.38	0.67	1.00E-04	2.30	1.45	7.50E-03
Leu	1.02	0.51	2.00E-03	1.49	0.89	5.70E-03
Met	0.10	0.65	7.31E-01	0.18	1.07	7.13E-01
Asn	0.32	0.53	2.08E-01	1.37	1.43	6.49E-02
Pro	0.58	0.40	1.24E-02	2.17	1.43	9.50E-03
Gln	0.19	0.73	5.73E-01	0.47	1.47	4.93E-01
Arg	3.25	1.23	4.00E-04	4.63	2.02	9.00E-04
Ser	0.14	0.37	4.28E-01	1.18	1.30	7.74E-02
Thr	1.20	0.45	4.00E-04	0.87	1.32	1.77E-01
Val	1.83	0.41	1.00E-04	1.15	0.90	2.10E-02
Trp	2.82	3.21	8.48E-02	4.87	1.51	1.00E-04
Tyr	1.67	0.67	5.00E-04	2.60	2.24	3.16E-02
